# Systematic Identification and Evolutionary Analysis of Catalytically Versatile Cytochrome P450 Monooxygenase Families Enriched in Model Basidiomycete Fungi

**DOI:** 10.1371/journal.pone.0086683

**Published:** 2014-01-22

**Authors:** Khajamohiddin Syed, Karabo Shale, Nataraj Sekhar Pagadala, Jack Tuszynski

**Affiliations:** 1 Department of Life Sciences, Faculty of Health and Environmental Sciences, Central University of Technology, Bloemfontein, Free State, South Africa; 2 Department of Chemical Engineering, University of Alberta, Edmonton, Alberta, Canada; 3 Department of Physics, University of Alberta, Edmonton, Alberta, Canada; University of Wisconsin - Madison, United States of America

## Abstract

Genome sequencing of basidiomycetes, a group of fungi capable of degrading/mineralizing plant material, revealed the presence of numerous cytochrome P450 monooxygenases (P450s) in their genomes, with some exceptions. Considering the large repertoire of P450s found in fungi, it is difficult to identify P450s that play an important role in fungal metabolism and the adaptation of fungi to diverse ecological niches. In this study, we followed Sir Charles Darwin’s theory of natural selection to identify such P450s in model basidiomycete fungi showing a preference for different types of plant components degradation. Any P450 family comprising a large number of member P450s compared to other P450 families indicates its natural selection over other P450 families by its important role in fungal physiology. Genome-wide comparative P450 analysis in the basidiomycete species, *Phanerochaete chrysosporium*, *Phanerochaete carnosa*, *Agaricus bisporus*, *Postia placenta*, *Ganoderma* sp. and *Serpula lacrymans*, revealed enrichment of 11 P450 families (out of 68 P450 families), CYP63, CYP512, CYP5035, CYP5037, CYP5136, CYP5141, CYP5144, CYP5146, CYP5150, CYP5348 and CYP5359. Phylogenetic analysis of the P450 family showed species-specific alignment of P450s across the P450 families with the exception of P450s of *Phanerochaete chrysosporium* and *Phanerochaete carnosa,* suggesting paralogous evolution of P450s in model basidiomycetes. P450 gene-structure analysis revealed high conservation in the size of exons and the location of introns. P450s with the same gene structure were found tandemly arranged in the genomes of selected fungi. This clearly suggests that extensive gene duplications, particularly tandem gene duplications, led to the enrichment of selective P450 families in basidiomycetes. Functional analysis and gene expression profiling data suggest that members of the P450 families are catalytically versatile and possibly involved in fungal colonization of plant material. To our knowledge, this is the first report on the identification and comparative-evolutionary analysis of P450 families enriched in model basidiomycetes.

## Introduction

Plant biomass is the most abundant source of photosynthetically fixed carbon on land and a renewable source for the sustainable production of biofuels, chemicals and materials [1]. It is composed of cellulose, hemicellulose and lignin. The main barrier in the utilization of cellulosic material is lignin, the most recalcitrant aromatic macromolecule [2] that protects cellulose and hemicellulose. In their natural environment fungi, lower eukaryotic organisms, developed an extraordinary ability to degrade and mineralize lignin. Among fungi, particularly white-rot basidiomycetes are the only fungi capable of complete mineralization of lignin [3]. Because of this enormous importance of basidiomycetes in carbon recycling, there has been an explosion in fungal genome sequencing projects undertaken in recent years.

The recent explosion of fungal genome sequencing projects, particularly regarding basidiomycete genomes, has revealed the presence of a large number of cytochrome P450 monooxygenases (P450s) in their genomes, with some exceptions [4]–[12]. P450s are heme-thiolate proteins ubiquitously distributed across the biological kingdom [13]. These enzymes perform a wide variety of reactions in stereo- and regio-selective manner [14] and their properties have been investigated for various pharmaceutical, biotechnological and environmental applications [15]–[18].

Genome sequencing of *Phanerochaete chrysosporium* [4], the first basidiomycete to be sequenced, revealed the presence of a large number of P450 genes (149 P450s and 10 pseudo-P450s) in its genome [4], [19]. Subsequent genome sequencing of different basidiomycete species exhibited the same phenomenon and showed a large contingent of P450s in their genomes. Brown rot basidiomycetes, *Postia placenta* [5], [20] and *Serpula lacrymans* [6] respectively had 190 and 164 P450s in their genomes. Genome sequencing of basidiomycete species [8] comprising six white-rots and five brown-rots revealed the presence of a large number of P450s, ranging from 126 to 249 P450s, in their genomes. The soft wood degrading white-rot basidiomycete *Phanerochaete carnosa* had 256 P450s in its genome, the largest P450 contingent among sequenced and annotated basidiomycetes [10]. *Ceriporiopsis subvermispora*, a selective lignin degrader, and *Agaricus bisporus*, a litter decomposer, showed the presence of 222 and 109 P450s in their genomes [7], [21]. Recent genome sequencing of polypore species showed the presence of 209 P450s in each *Phlebia brevispora* and *Ganoderma* sp. and 199 P450s in *Bjerkandera adusta* [11], [12]. The medicinal mushroom *Ganoderma lucidum* had 198 P450s in its genome [9].

The presence of a large number of P450s in basidiomycete genomes clearly suggests that these P450s play an important role(s) in fungal metabolism [22]–[24]. P450s were found to play an essential role in many pathways in the primary and secondary metabolism of fungal species, including membrane ergosterol biosynthesis [25], [26], outer spore wall components biosynthesis [27], alkane and fatty acids degradation [28], fatty acids hydroxylation [29], mycotoxins (i.e. aflatoxins, trichothecenes, and fumonisins) [30]–[32] and plant hormones biosynthesis (gibberellin biosynthesis) [33]. P450s from basidiomycete fungi were found to be functionally diverse and showed oxidization of a range of substrates including different classes of xenobiotic compounds [20], [34]–[42]. Recently, a catalytically versatile P450 displaying extraordinary substrate oxidation capability was found in the model white rot basidiomycete, *Phanerochaete chrysosporium* [37]. The P450, belonging to the CYP63 family, was found to oxidize a range of xenobiotic compounds compared to P450s across the biological kingdoms (bacteria and animals) [37].

Identification of specific P450s from a large contingent of P450s playing an important role in fungal metabolism and fungal adaptation, such as colonization of wood, is necessary in order to engineer fungal organisms for efficient degradation/bioconversion of wood into human valuables. In this study, we followed Sir Charles Darwin’s theory of natural selection to identify such P450s in the selected basidiomycete species, *Phanerochaete chrysosporium*, *Phanerochaete carnosa*, *Agaricus bisporus*, *Postia placenta*, *Ganoderma* sp. and *Serpula lacrymans*, representative of preference for different plant material/components degradation (Table 1). Furthermore, we carried out comparative evolutionary analysis and functional analysis of P450s to understand the molecular basis for selective enrichment of P450 families in the selected basidiomycetes.

**Table 1:** Typical characteristics of selected model basidiomycetes representative of preferring different plant material/components degradation.

Name of the basidiomycete	Type of fungi	Well-known for	Substrate preference	Preference of degradation of plant component	P450 count	References
*Phanerochaete chrysosporium*	White rot	Model fungus for understanding the mechanism of wood degradation	Hardwood	Efficiently degrades all plant cell wall components (lignin, cellulose and hemicellulose)	149	4, 19, 41
*Phanerochaete carnosa* HHB-10118-Sp.	White rot	Model fungus for understanding the mechanism of softwood degradation	Softwood (coniferous wood)	Efficiently degrades all plant cell wall components (lignin, cellulose and hemicellulose)	266	10
*Agaricus bisporus var bisporus* (H97)	–	Model fungus for understanding the adaptation, persistence, and growth in the humic-rich leaf litter environment. Premier cultivated button mushroom.	Leaf and litter	Non-wood secondary decomposer, leaf and needle litter degrader	109	21, 44
*Ganoderma* sp. 10597 SS1 v1.0/*Ganoderma lucidum* strain 260125-1	White rot	Shelf mushrooms or bracket fungi/model fungus for study of the biosynthesis of natural medicines	Softwood and hardwood	Capable of removing plant cell wall components, either lignin only (selective delignification) or both lignin and cellulose	209/198	9, 11,12
*Postia placenta* MAD 698-R	Brown rot	Model brown rot fungus used for study of wood degradation	Hardwood	Primarily degrades plant cell wall component cellulose and hemicellulose	190	5, 20
*Serpula lacrymans* S7.3	Brown rot (dry rot)	Model system to study evolutionary processes and life history traits in basidiomycetes. Well known as dry rot, destroyer of indoor wood construction,	Dry hardwood	Primarily degrades plant cell wall component cellulose and hemicellulose	164	6, 44
**Out-group fungi (non-wood-degraders)**						
*Tremella mesenterica*	Golden jelly fungus	Mycoparasite	–	–	8	8,44
*Cryptococcus neoformans*	Opportunistic pathogen	Animal pathogen/parasite	–	–	10	46,52

As an out-group, the non-wood-degrading basidiomycete species, *Tremella mesenterica* (mycoparasite) and *Cryptococcus neoformans* (animal pathogen/parasite) are also shown in the table. *Phanerochaete carnosa* has been exclusively isolated from softwood (10) has been represented as model basidiomycete for understanding the mechanism of softwood degradation. In comparison to *Ganoderma lucidum*, the model medicinal mushroom (9), *Ganoderma* sp. proved to have an additional 10 sub-families in its genome (12). For this reason, in this study we used *Ganoderma* sp. P450s as representative of *Ganoderma lucidum*.

## Materials and Methods

### Fungal Genome Databases

Publicly available genomes of *Phanerochaete chrysosporium* [4], *Phanerochaete carnosa* [10], *Agaricus bisporus* [21], *Ganoderma* sp. [11], *Postia placenta* [5] and *Serpula lacrymans* [6] were accessed in the genome database of the Joint Genome Institute (JGI) of the US Department of Energy (US-DOE) (http://genome.jgi.doe.gov/programs/fungi/index.jsf) [43]. The basidiomycete strains and databases used in this study were *Phanerochaete chrysosporium* v2.0: http://genome.jgi-psf.org/Phchr1/Phchr1.home.html; *Phanerochaete carnosa* HHB-10118-Sp v1.0: http://genome.jgi-psf.org/Phaca1/Phaca1.home.html; *Agaricus bisporus var bisporus* (H97) v2.0: http://genome.jgi.doe.gov/Agabi_varbisH97_2/Agabi_varbisH97_2.home.html; *Ganoderma* sp. 10597 SS1 v1.0: http://genome.jgi-psf.org/Gansp1/Gansp1.home.html; *Postia placenta* MAD 698-R v1.0: http://genome.jgi.doe.gov/Pospl1/Pospl1.home.html; *Serpula lacrymans* S7.9 v2.0: http://genome.jgi.doe.gov/SerlaS7_9_2/SerlaS7_9_2.home.html.

### Genome-wide P450 Analysis

P450 monooxygenases of selected basidiomycete species were obtained from the published data and publicly available data bases. The literature that was consulted on the respective species included the following publications: *Phanerochaete chrysosporium*: Syed and Yadav [19] and Hirosue et al., [41]; *Phanerochaete carnosa*: Sukuzi et al., [10]; *Postia placenta*: Ide et al., [20] and *Ganoderma* sp.: Syed et al., [12]. Annotated P450s for *Agaricus bisporus*, *Serpula lacrymans* and *Tremella mesenterica* were downloaded from the Fungal Cytochrome P450 Database (FCPD) (http://p450.riceblast.snu.ac.kr/index.php?a=view) [44]. The revised FCPD represents fungal P450 nomenclature equivalent to the standard P450 nomenclature [45]. P450s for the medicinal mushroom *Ganoderma lucidum* strain 260125-1 [9] were kindly provided by Dr David Nelson, University of Tennessee, USA. The cytochrome P450 webpage (http://drnelson.uthsc.edu/P450seqs.dbs.html) [46] was also visited for analysis of P450 signature domains and confirmation of pseudo P450s. Furthermore, the annotated P450s of animal pathogen/parasite *Cryptococcus neoformans* were downloaded from the cytochrome P450 webpage [46].

Among the P450 sequences resourced, as mentioned above, only the authentic P450s, i.e. those P450s containing both P450 signature motifs (heme-binding sequence motif FXXGXXXCXG and the EXXR motif in the K-helix), were selected for analysis. Hence, the P450 count reported in this study for the P450 family is slightly different from the P450 count presented in published literature and public data bases (Table S1). Pseudo P450s and alleles (for *Postia placenta*) are not included in this study.

### Criteria for Selection of Enriched P450 Family

Selected basidiomycete species showed a large number of P450 genes in their genome (Table 1). A total of 1061 P450s were found in six basidiomycetes. The 1061 P450s were grouped under different families (Table S1). We set a 2% cut-off P450 count (∼21 P450s) in the total number of P450s (1061 P450s) for a P450 family to be considered as a P450 family enriched across the selected basidiomycete species (Table S1).

### Evolutionary Analysis

P450 protein sequences of the enriched family were subjected to phylogenetic analysis using the Molecular Evolutionary Genetics Analysis (MEGA 5.05) software [47]. Phylogenetic analysis was inferred using the minimum evolution method [48]. The evolutionary distances were computed using the Poisson correction method [49] and are in the units of the number of amino acid substitutions per site. The minimum evolution tree was searched using the close-neighbor-interchange algorithm [50] at a search level of 1. The neighbor-joining algorithm [51] was used to generate the initial tree.

The minimum evolution method generates trees that show pairing of the true neighbor taxons (e.g. proteins) and reflect the true evolutionary aspect, as this method chooses the smallest value of the sum of all branches as an estimate of the tree. This method has also been widely used in P450 research for phylogenetic analysis of P450s [9]. Considering its accuracy and wide use in P450 research, in this study we used the minimum evolution method for phylogenetic analysis of P450s.

### Gene-structure Analysis

The physical location of P450 genes on a particular scaffold was identified by searching the JGI fungal genome database using the P450 protein ID. Analysis of exon-intron organization (in order to identify ortholog and paralog P450s) was carried out as follows: P450 gene structure graphics downloaded from JGI was aligned in such a way that exons with the same size would align together. Untranslated regions were manually curated for proper alignment of exons and introns. Parallel lines representing gene size and vertical lines representing introns were drawn as per gene size and intron location described in the JGI data base. P450s whose gene structures are similar are presented in the figures. The gene structure of P450s whose gene structure is not similar to any of the P450s in the family (within and across the species) is presented with the numbers of exons and introns using numerical notation. Furthermore, manually analyzed intron-exon organization was verified by the Spidey mRNA-to-genomic alignment programme (http://www.ncbi.nlm.nih.gov/spidey/).

## Results and Discussion

In order to identify P450 families enriched across basidiomycetes, we selected six basidiomycete species representative of preference for different plant material/components degradation (Table 1). The model white rot basidiomycete *Phanerochaete chrysosporium* represents basidiomycete with the capability of hard wood degradation and complete mineralization of all components of plant cell wall material (lignin, cellulose and hemicellulose) [4]. *Phanerochaete carnosa* represents soft wood (particularly coniferous wood) degrading white rot fungi with capabilities similar to *Phanerochaete chrysosporium* [10]. *Ganoderma* sp. represents white rot fungus with a selective lignin degradation capability and able to grow both soft and hard wood [11]. In comparison to *Ganoderma lucidum*, the medicinal mushroom [9], *Ganoderma* sp. [12] proved to have an additional 10 sub-families in its genome. For this reason, in this study we used *Ganoderma* sp. P450s as representative of *Ganoderma* strains. *Agaricus bisporus*, an edible button mushroom fungus, is a non-wood-degrading fungus and considered as model fungus for studying adaptation and growth in humic-rich leaf litter environments [21]. *Postia placenta* and *Serpula lacrymans* represent brown-rots with the capability of primarily removing cellulose and hemicellulose [5], [6]. However, *Serpula lacrymans* is evolutionarily divergent compared to *Postia placenta* and well known for causing dry-rot in wood [6]. The non-wood-degrading basidiomycetes, *Tremella mesenterica* (mycoparasite) [8] and *Cryptococcus neoformans* (animal pathogen/parasite) [52] were used as an out-group in this study (Table 1).

### Genome-wide Comparative Analysis and Identification of P450 Families Enriched in Model Basidiomycetes

**Figure 1:**
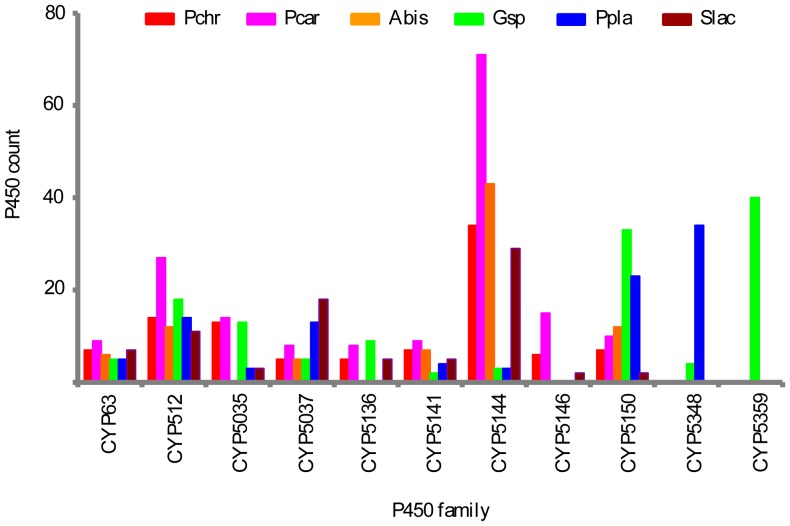
Identification and comparative analysis of P450 families enriched in model basidiomycete fungi. Model basidiomycetes, *Phanerochaete chrysosporium* (*Pchr*), *Phanerochaete carnosa* (Pcar), *Agaricus bisporus* (Abis), *Ganoderma* sp. (Gsp) as representative of *Ganoderma lucidum*, *Postia placenta* (Ppla) and *Serpula lacrymans* (Slac), showing preference for degradation of different plant material/components (Table 1) were selected for identification of P450 families enriched in their genomes.

In total 1061 P450s were found in the selected six basidiomycete species (Table S1). The P450 count in fungal species varied from 149 to 266, with the two white-rots representing the lowest (*Phanerochaete chrysosporium*) and the highest (*Phanerochaete carnosa*) number of P450s in their genomes (Table 1). The 1061 P450s found in the basidiomycete species, *Phanerochaete chrysosporium*, *Phanerochaete carnosa*, *Ganoderma* sp. *Agaricus bisporus*, *Postia placenta*, and *Serpula lacrymans,* were grouped into 68 P450 families (Table S1). Based on the 2% cut-off criterion to consider a P450 family to be an enriched family, we identified nine P450 families, CYP63, CYP512, CYP5035, CYP5037, CYP5135, CYP5141, CYP5144, CYP5146, and CYP5150, that were enriched across the selected basidiomycete species and two P450 families, CYP5348 and CYP5359, that were enriched in species-specific manner (Figure 1). Among the enriched families, CYP5144 family showed the highest number of P450s (183 P450s), followed by families CYP512 (96 P450s), CYP5150 (87 P450s), CYP5037 (54 P450s), CYP5035 (46 P450s), CYP5359 (40 P450s), CYP5348 (38 P450s), CYP5141 (34 P450s) and CYP5136 (27 P450s). Analysis of enriched P450 family conservation across the selected basidiomycetes revealed the absence of the CYP5035 family in *Agaricus bisporus*. The CYP5136 family was not found in *Agaricus bisporus* and *Postia placenta* and the CYP5146 family is missing in *Agaricus bisporus*, *Ganoderma* sp. and *Postia placenta* (Figure 1). The CYP5359 family was found only in *Ganoderma* sp. Furthermore, *Ganoderma* sp. and *Postia placenta* are the only representatives of the CYP5348 family.

The selected six basidiomycete species, *Phanerochaete chrysosporium*, *Phanerochaete carnosa*, *Ganoderma* sp. *Agaricus bisporus*, *Postia placenta*, and *Serpula lacrymans,* showed a high variation in P450 count in enriched P450 families (Fig. 1 and Table S1), with the exception of the CYP63 family. The P450 count for the CYP63 family among the selected fungi ranged from 5 to 9 P450s. For the CYP5150 family, *Ganoderma* sp. and *Postia placenta* showed the highest number of P450s (33 and 23 P450s, respectively) compared to the rest of the basidiomycetes that showed two to 12 P450s. In contrast to the P450 count observed for the CYP5150 family, *Ganoderma* sp. and *Postia placenta* showed the lowest number of P450s (three P450s each) in the CYP5144 family. The P450 count for the CYP5144 family among the rest of the four model basidiomycetes varied from 29 to 71; the highest number of CYP5144 member P450s was found in *Phanerochaete carnosa*. For the CYP5037 family the highest number of member P450s was found in model brown and dry-rots, *Postia placenta* (13 P450s) and *Serpula lacrymans* (18 P450s). The P450 family CYP5359 is exclusively present and highly enriched (40 P450s) in *Ganoderma* sp. The CYP5348 family is highly enriched in *Postia placenta* and only four CYP5348 family member P450s were found in *Ganoderma* sp. Furthermore, our analysis showed the absence of 11 enriched P450 families of plant-material-degrading basidiomycetes in the non-wood-degrading basidiomycetes, *Tremella mesenterica* and *Cryptococcus neoformans* (Table S2). It is noteworthy that the non-wood-degrading basidiomycete fungus *Tremella mesenterica* and *Cryptococcus neoformans* showed low copies of P450s (8 and 10) in their genomes (Table S2).

High conservation of a few P450 families across the fungi has been well documented [22]. The CYP61 and CYP51 P450 families are highly conserved across the fungi, whereas the CYP53 and CYP505 families are fairly conserved. However, to our knowledge, comparative analysis of P450s at a family level in six model basidiomycetes representative of diverse plant material/components degradation (Table 1) has not been reported before. Hence, our study constitutes the first report on comparative P450 analysis in six model basidiomycetes and also represents an identification of enriched P450 families in the model basidiomycetes.

Enrichment of 11 P450 families in the model basidiomycete species suggests that the member P450s of the enriched family play an important role in fungal metabolism and possibly fungal adaptation to diverse ecological niches.

### Extensive P450 Gene Duplications Led to Selective P450 Family Enrichment in Model Basidiomycetes

**Figure 2:**
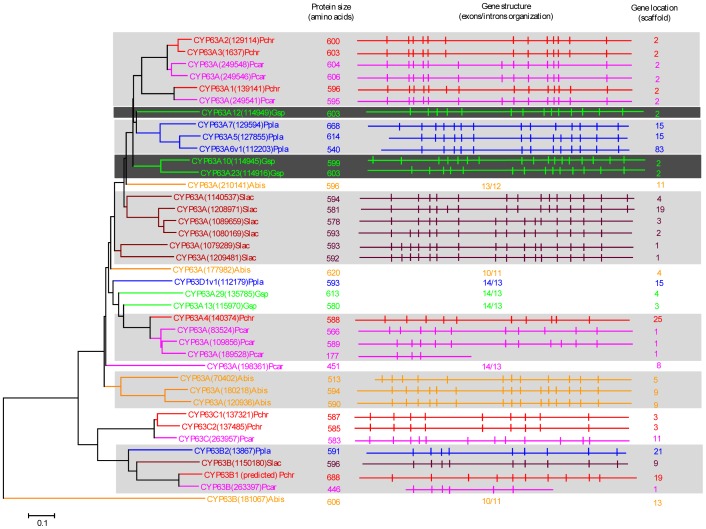
Phylogenetic and gene-structure analysis of CYP63 family. In total 38 CYP63 P450 sequences from six selected model basidiomycetes (Fig. 1) were included in the tree. The minimum evolution tree was constructed using the close-neighbor-interchange algorithm in MEGA (version 5.05). For easy of visual identity, the tree branch color, protein name, protein ID (parenthesis) and model basidiomycete species name were presented with unique color. The protein size in amino acids is also shown in the figure. Gene-structure analysis for each P450 was presented in the form of exon-intron organization. A graphical format showing parallel (gene size) and vertical lines (introns) is presented for P450s showing similar gene structure (highlighted with unique background color). For the rest of the P450s the number of exons and introns was shown. The genetic location of P450 is shown in the form of the scaffold number. Abbreviations: Pchr, *Phanerochaete chrysosporium*; Pcar, *Phanerochaete carnosa*; Abis, *Agaricus bisporus*; Gsp, *Ganoderma* sp.; Ppla, *Postia placenta*; Slac, *Serpula lacrymans*.

**Figure 3:**
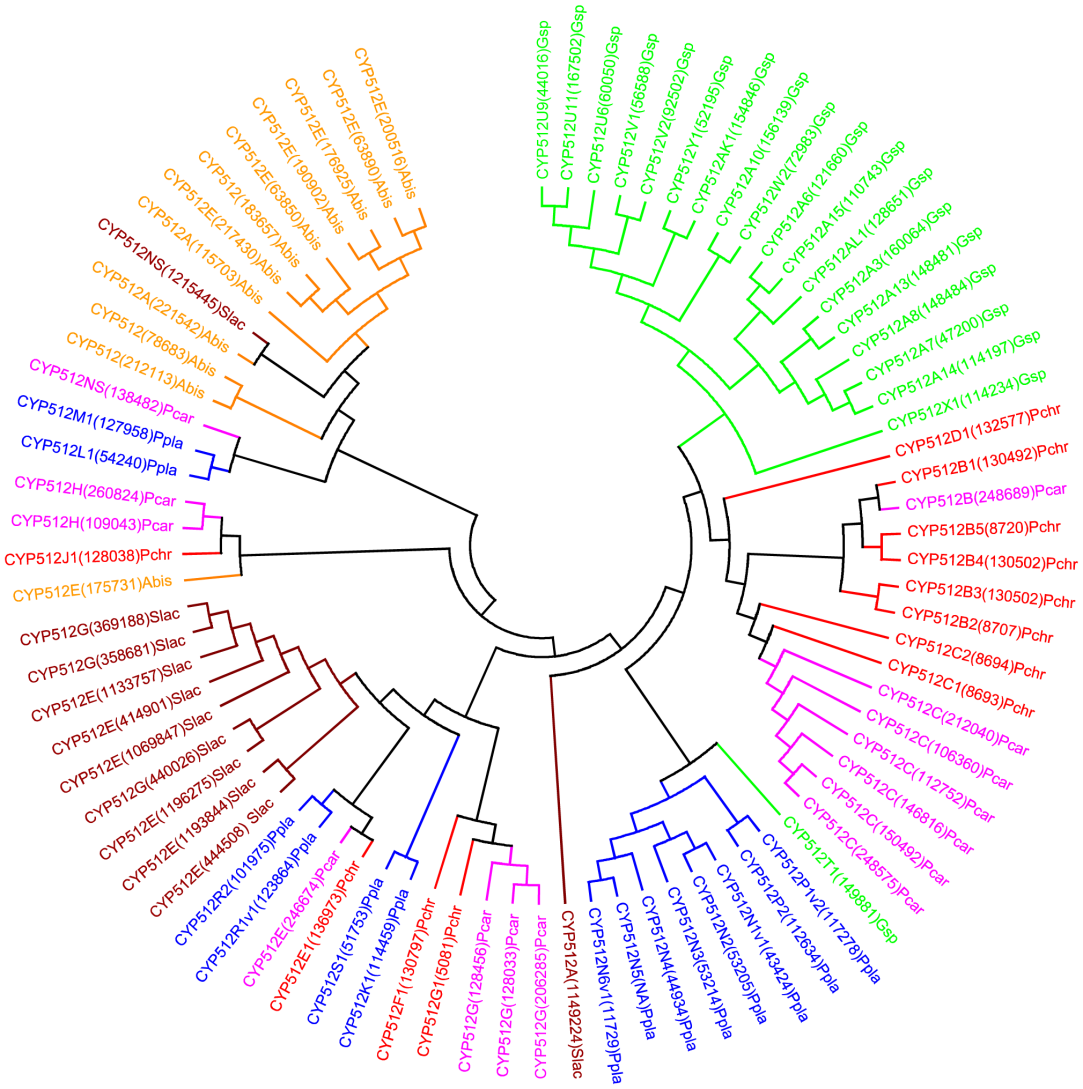
Minimum evolution tree of CYP512 family. In total 82 CYP512 P450 sequences were included in the tree. The tree was constructed using the close-neighbor-interchange algorithm in MEGA (version 5.05). For ease of visual identity, the tree branch color, protein name, protein ID (parenthesis) and species name were presented with unique color. Abbreviations: Pchr, *Phanerochaete chrysosporium*; Pcar, *Phanerochaete carnosa*; Abis, *Agaricus bisporus*; Gsp, *Ganoderma* sp.; Ppla, *Postia placenta*; Slac, *Serpula lacrymans*.

**Figure 4:**
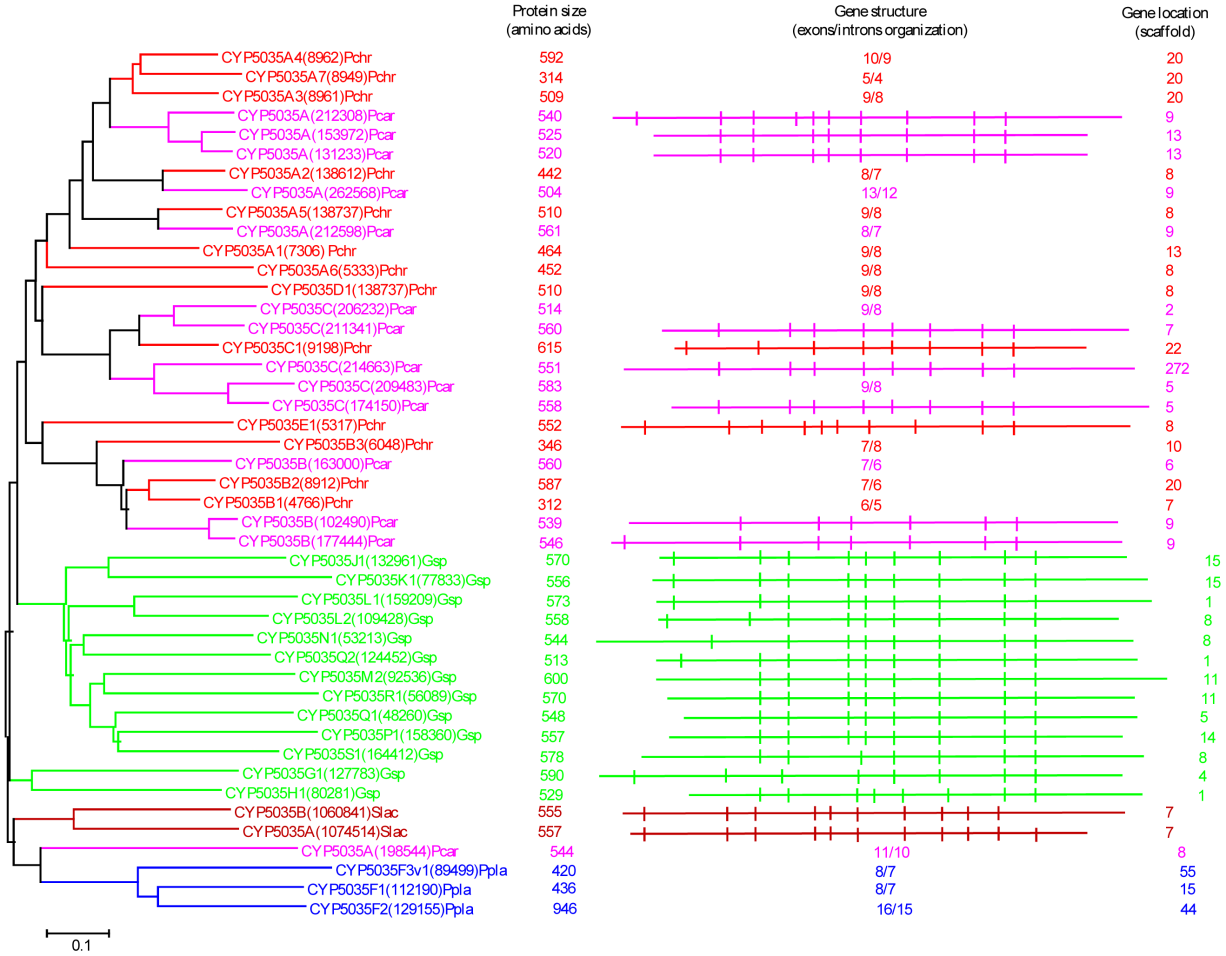
Phylogenetic and gene-structure analysis of CYP5035 family. In total 45 CYP5035 P450 sequences from five model basidiomycetes (Fig. 1) were included in the tree. The minimum evolution tree was constructed using the close-neighbor-interchange algorithm in MEGA (version 5.05). For ease of visual identity, the tree branch color, protein name, protein ID (parenthesis) and model basidiomycete species name were presented with unique color. The protein size in amino acids is also shown in the figure. The gene-structure analysis for each P450 was presented in the form of exon-intron organization. A graphical format showing parallel (gene size) and vertical lines (introns) is presented for P450s showing similar gene structure. For the rest of the P450s, the number of exons and introns was shown. The genetic location of P450 is shown in the form of the scaffold number. Abbreviations: Pchr, *Phanerochaete chrysosporium*; Pcar, *Phanerochaete carnosa*; Abis, *Agaricus bisporus*; Gsp, *Ganoderma* sp.; Ppla, *Postia placenta*; Slac, *Serpula lacrymans*.

**Figure 5:**
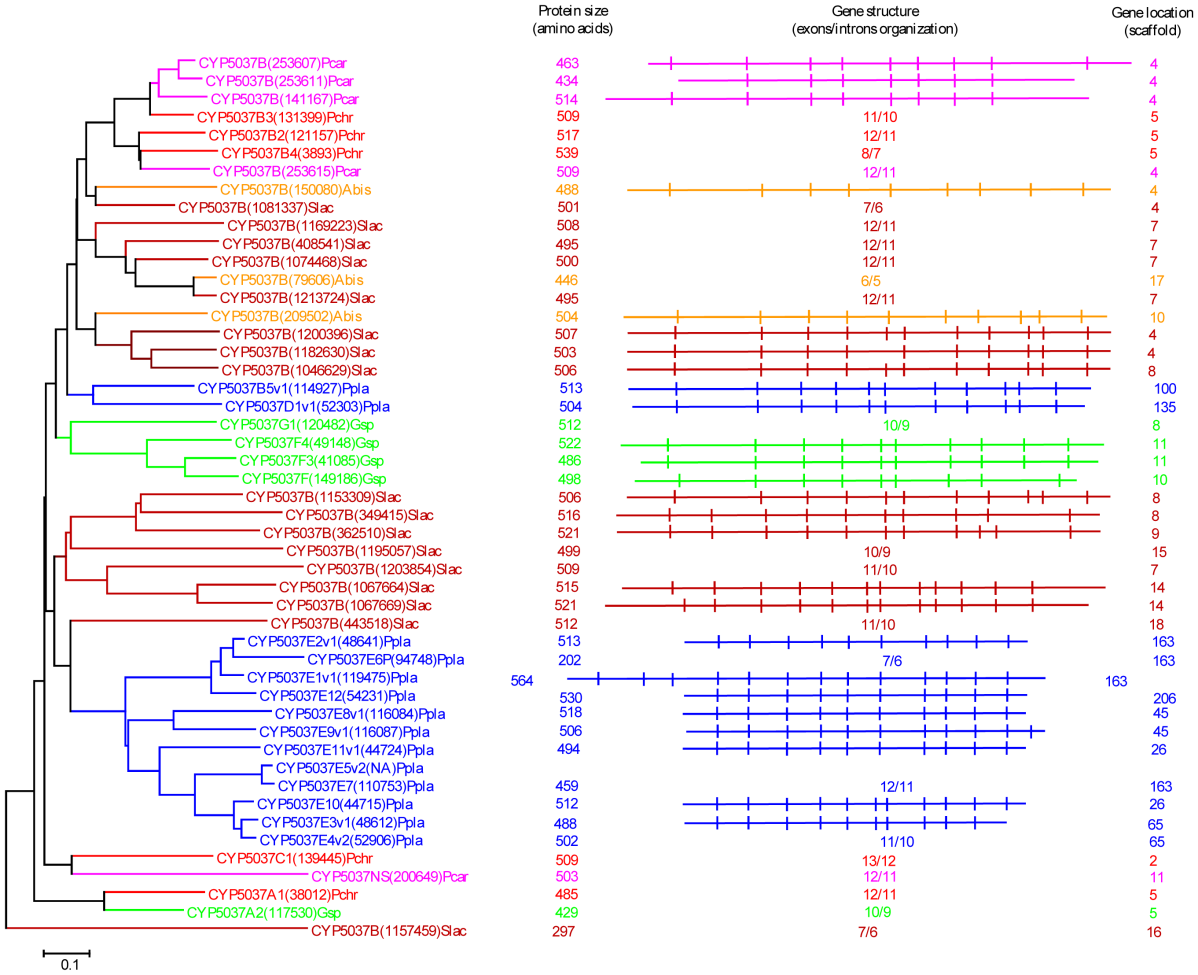
Phylogenetic and gene-structure analysis of CYP5037 family. In total 49 CYP5037 P450 sequences from six model basidiomycetes (Fig. 1) were included in the tree. The minimum evolution tree was constructed using the close-neighbor-interchange algorithm in MEGA (version 5.05). For ease of visual identity, the tree branch color, protein name, protein ID (parenthesis) and model basidiomycete species name were presented with unique color. The protein size in amino acids is also shown in the figure. The gene-structure analysis for each P450 was presented in the form of exon-intron organization. A graphical format showing parallel (gene size) and vertical lines (introns) is presented for P450s showing similar gene structure. For the rest of the P450s, the number of exons and introns was shown. The genetic location of P450 is shown in the form of the scaffold number. Abbreviations: Pchr, *Phanerochaete chrysosporium*; Pcar, *Phanerochaete carnosa*; Gsp, *Ganoderma* sp.; Ppla, *Postia placenta*; Slac, *Serpula lacrymans*.

**Figure 6:**
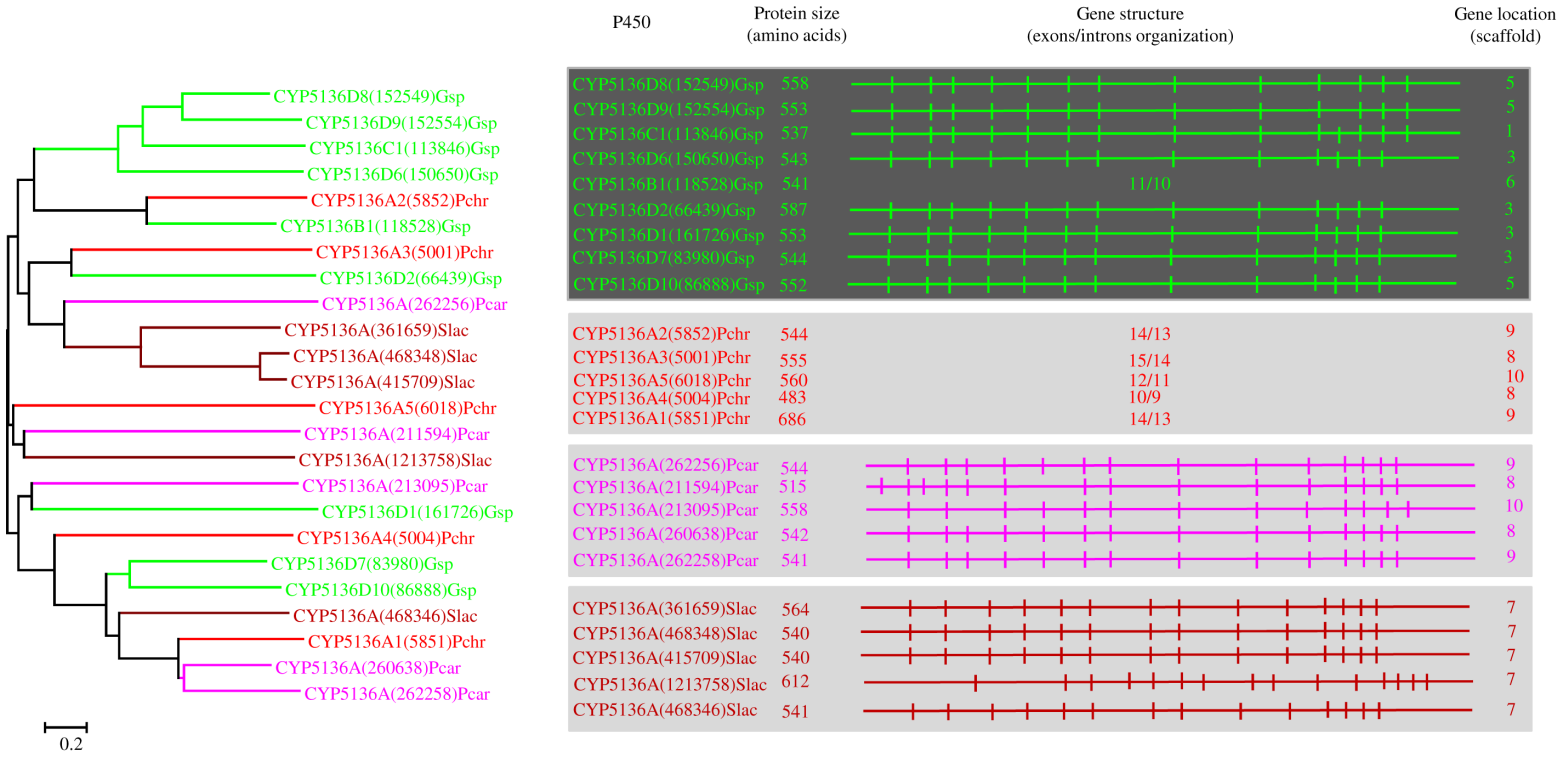
Phylogenetic and gene-structure analysis of CYP5136 family. Twenty-four P450 sequences from four model basidiomycetes *Phanerochaete chrysosporium* (Pchr), *Phanerochaete carnosa* (Pcar), *Ganoderma* sp. (Gsp) and *Serpula lacrymans* (Slac) were included in the tree. A minimum evolution tree was constructed using the close-neighbor-interchange algorithm in MEGA (version 5.05). For ease of visual identity, the tree branch color, protein name, protein ID (parenthesis) and model basidiomycete species name were presented in red (Pchr) and pink (Pcar). The protein size in amino acids is also shown in the figure. Gene-structure analysis for each P450 was presented in the form of exon-intron organization. A graphical format showing parallel (gene size) and vertical lines (introns) is presented for P450s showing similar gene structure (highlighted with unique background color). For the rest of the P450s, the number of exons and introns was shown. The genetic location of P450 is shown in the form of the scaffold number.

**Figure 7:**
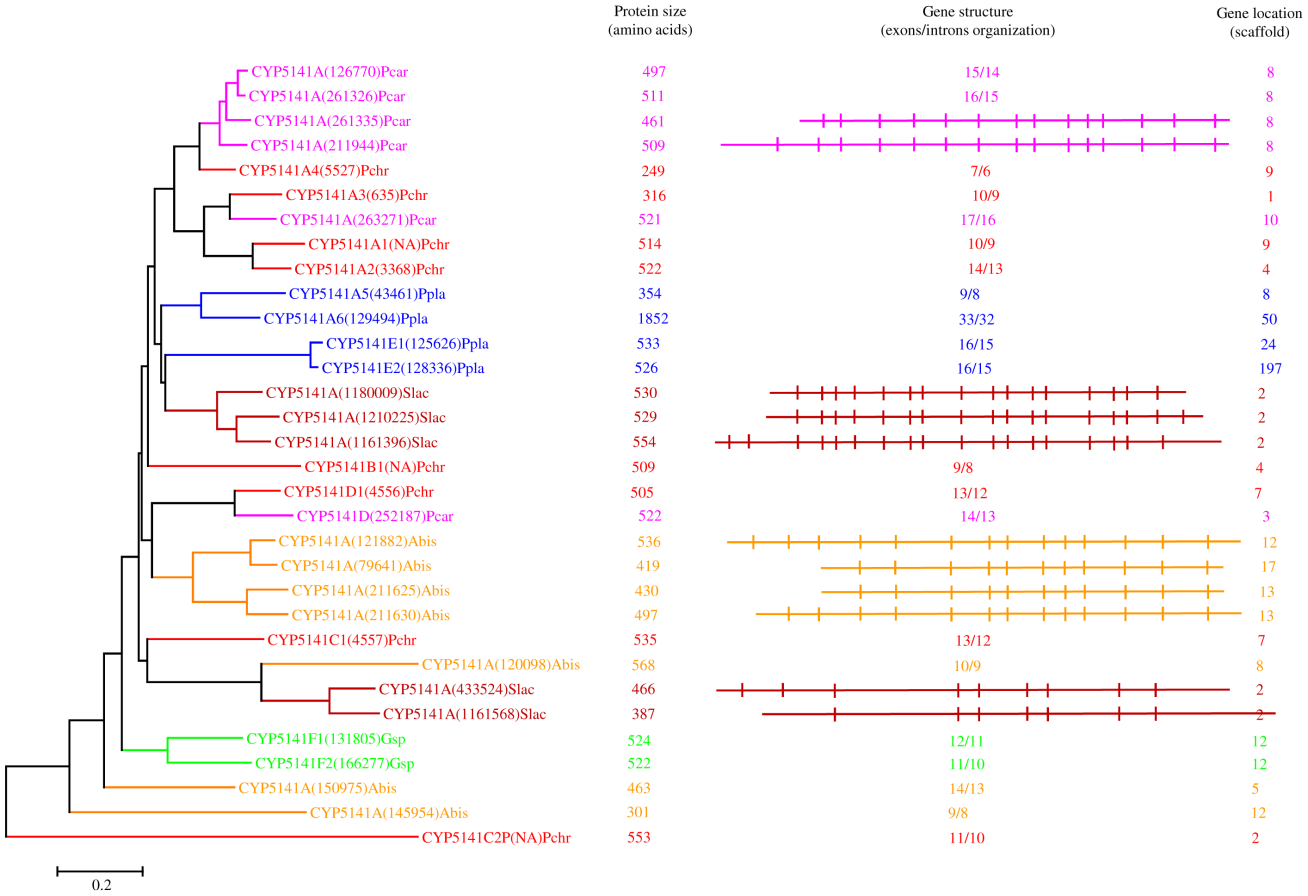
Phylogenetic and gene-structure analysis of CYP5141 family. Thirty-two CYP5141 P450 sequences from six model basidiomycetes (Fig. 1) were included in the tree. The minimum evolution tree was constructed using the close-neighbor-interchange algorithm in MEGA (version 5.05). For ease of visual identity, the tree branch color, protein name, protein ID (parenthesis) and model basidiomycete species name were presented with unique color. The protein size in amino acids is also shown in the figure. The gene-structure analysis for each P450 was presented in the form of exon-intron organization. A graphical format showing parallel (gene size) and vertical lines (introns) is presented for P450s showing similar gene structure. For the rest of the P450s, the number of exons and introns was shown. The genetic location of P450 is shown in the form of the scaffold number. Abbreviations: Pchr, *Phanerochaete chrysosporium*; Pcar, *Phanerochaete carnosa*; Abis, *Agaricus bisporus*; Gsp, *Ganoderma* sp.; Ppla, *Postia placenta*; Slac, *Serpula lacrymans*.

**Figure 8:**
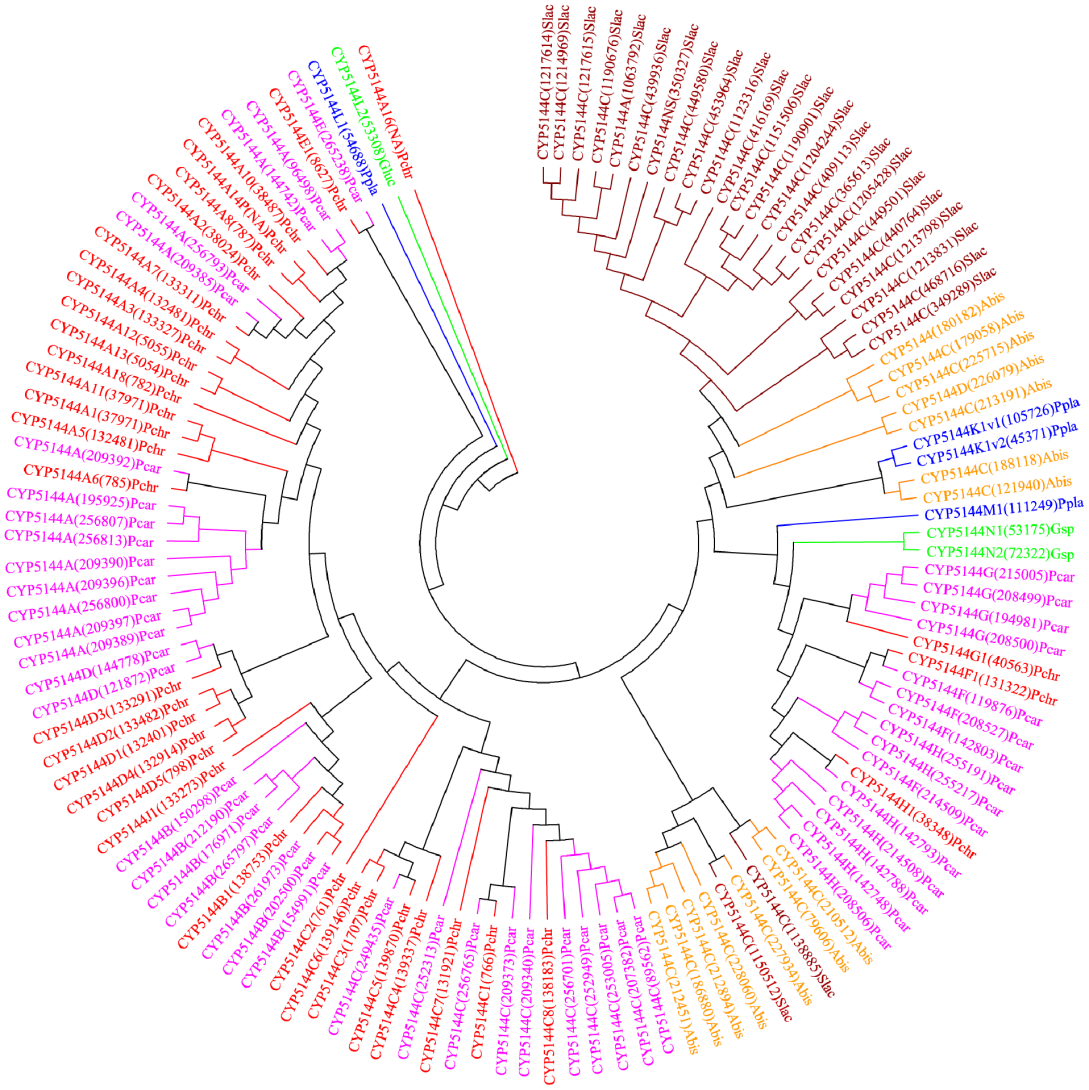
Phylogenetic analysis of CYP5144 family. In total 128 CYP5144 P450 sequences from six model basidiomycetes (Fig. 1) were included in the tree. The minimum evolution tree was constructed using the close-neighbor-interchange algorithm in MEGA (version 5.05). For ease of visual identity, the tree branch color, protein name, protein ID (parenthesis) and model basidiomycete species name were presented with unique color. Abbreviations: Pchr, *Phanerochaete chrysosporium*; Pcar, *Phanerochaete carnosa*; Abis, *Agaricus bisporus*; Gsp, *Ganoderma* sp.; Ppla, *Postia placenta*; Slac, *Serpula lacrymans*.

**Figure 9:**
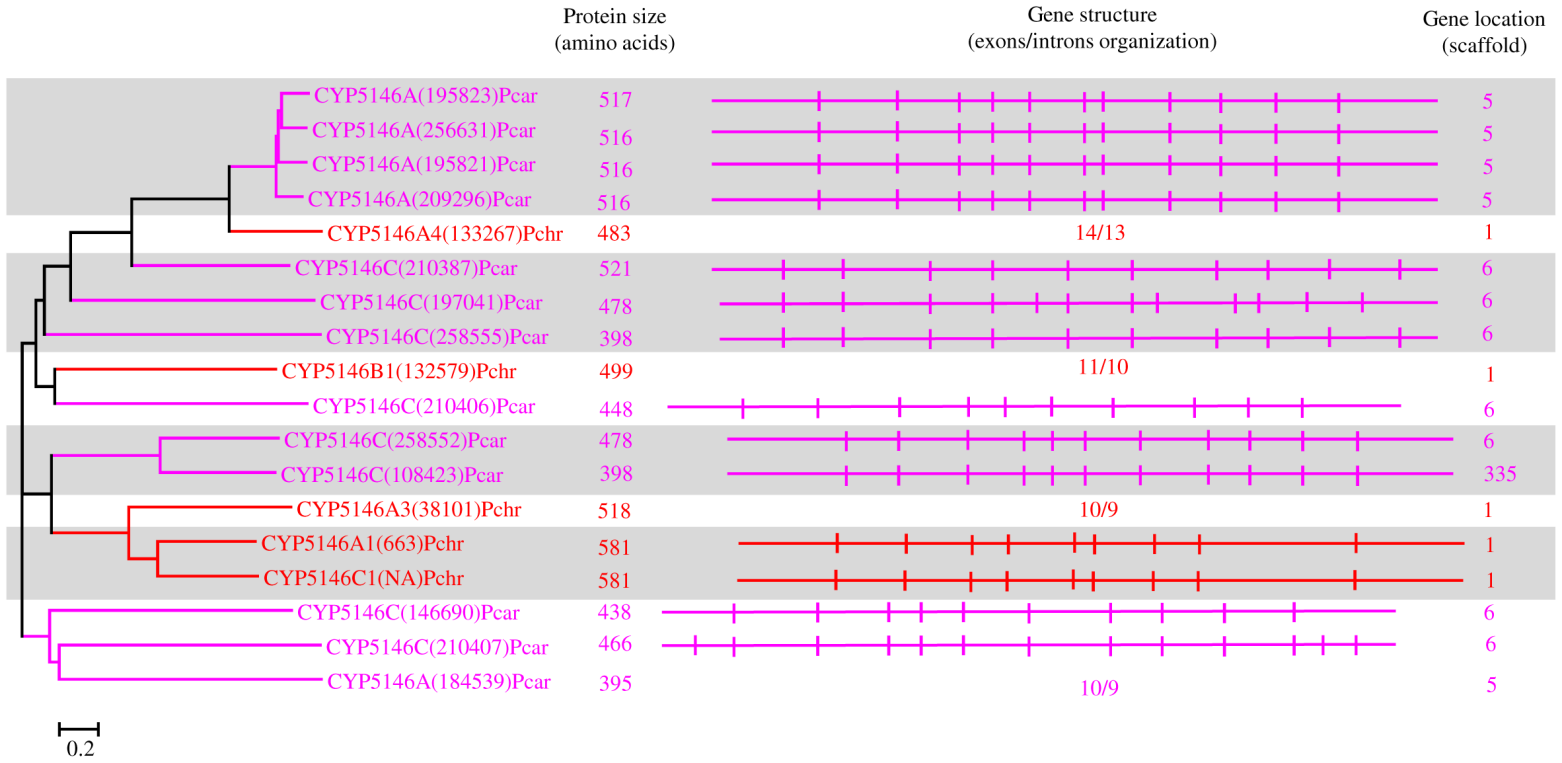
Phylogenetic and gene-structure analysis of CYP5146 family. Eighteen P450 sequences from two white rot basidiomycetes, *Phanerochaete chrysosporium* (Pchr) and *Phanerochaete carnosa* (Pcar), were included in the tree. The minimum evolution tree was constructed using the close-neighbor-interchange algorithm in MEGA (version 5.05). For ease of visual identity, the tree branch color, protein name, protein ID (parenthesis) and model basidiomycete species name were presented in red (Pchr) and pink (Pcar). The protein size in amino acids is shown in the figure. The gene-structure analysis for each P450 was presented in the form of exon-intron organization. A graphical format showing parallel (gene size) and vertical lines (introns) is presented for P450s showing similar gene structure (highlighted with unique color). For the rest of the P450s, the number of exons and introns was shown. The genetic location of P450 is shown in the form of the scaffold number.

**Figure 10:**
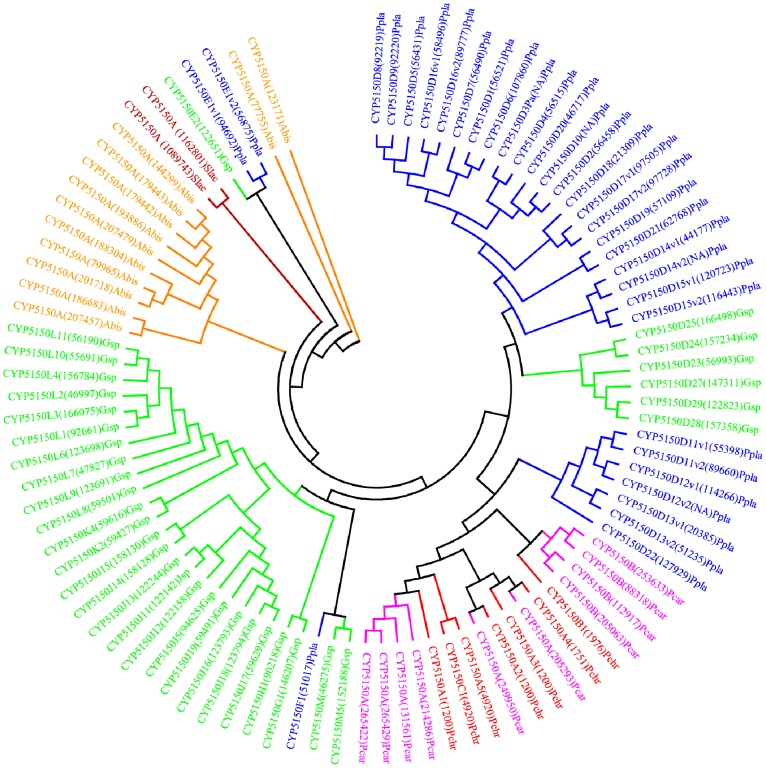
Phylogenetic analysis of CYP5150 family. In total 96 CYP5150 P450 sequences from six model basidiomycetes (Fig. 1) were included in the tree. The minimum evolution tree was constructed using the close-neighbor-interchange algorithm in MEGA (version 5.05). For ease of visual identity, the tree branch color, protein name, protein ID (parenthesis) and model basidiomycete species name were presented with unique color. Abbreviations: Pchr, *Phanerochaete chrysosporium*; Pcar, *Phanerochaete carnosa*; Abis, *Agaricus bisporus*; Gsp, *Ganoderma* sp.; Ppla, *Postia placenta*; Slac, *Serpula lacrymans*.

**Figure 11:**
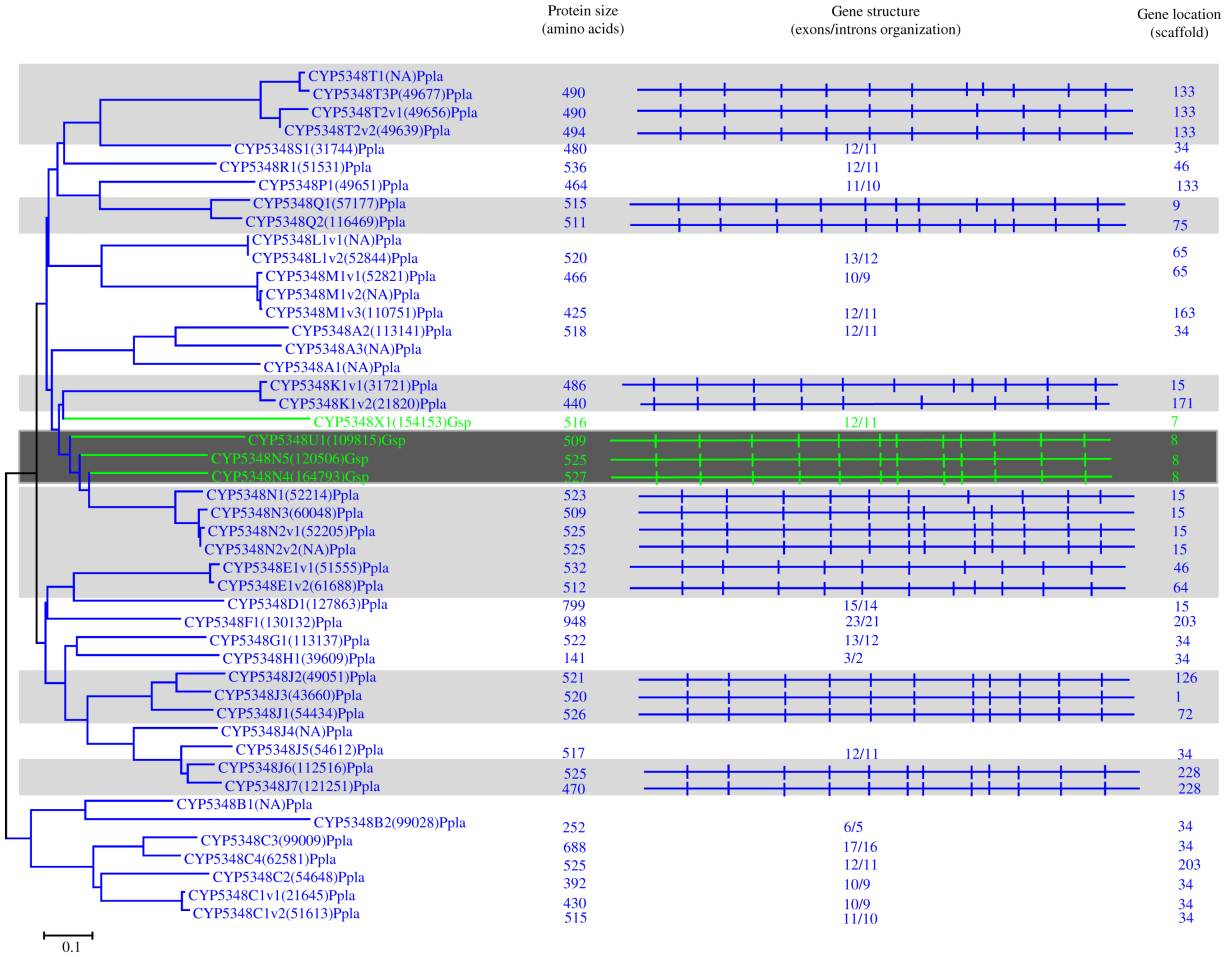
Phylogenetic and gene-structure analysis of CYP5348 family. In total 47 CYP5348 P450 sequences from two model basidiomycetes, *Postia placenta* (Ppla) and *Ganoderma* sp. (Gsp), were included in the tree. The minimum evolution tree was constructed using the close-neighbor-interchange algorithm in MEGA (version 5.05). For ease of visual identity, the tree branch color, protein name, protein ID (parenthesis) and model basidiomycete species name were presented in blue (Ppla) and magenta (Gsp). The protein size in amino acids is shown in the figure. The gene-structure analysis for each P450 was presented in the form of exon-intron organization. A graphical format showing parallel (gene size) and vertical lines (introns) is presented for P450s showing similar gene structure. For the rest of the P450s, the number of exons and introns was shown. The genetic location of P450 is shown in the form of the scaffold number.

Our genome-wide comparative analysis of P450s in selected model basidiomycetes revealed enrichment of 11 P450 families (Fig. 1). In order to gain insight into the evolution of these P450 families, we performed phylogenetic analysis and gene-structure analysis on member P450s that belong to the enriched P450 family. Phylogenetic analysis of 11 P450 families (Figures 2–11) revealed grouping of species-specific P450s in each family with the exception of P450s from *Phanerochaete* species. Joint alignment of *Phanerochaete chrysosporium* and *Phanerochaete carnosa* P450s in nine P450 families (Figures 2–10) suggested that P450s in both white-rots have high homology compared to P450s from other model basidiomycetes. Conservation of high homolog P450s in both *Phanerochaete* species fits perfectly with the well-known fact that *Phanerochaete chrysosporium* and *Phanerochaete carnosa* are closely related and evolved by a recent divergence to adapt to different ecological niches. In each P450 family, a few P450 proteins (Figures 2–10) were found aligned with P450s from different basidiomycete species, indicating that these P450s possibly descended from a common origin before specification (orthologs). Species-specific alignment of P450s in P450 families enriched in basidiomycetes clearly suggests that these P450s are paralogs and possibly evolved by gene duplications.

**Figure 12:**
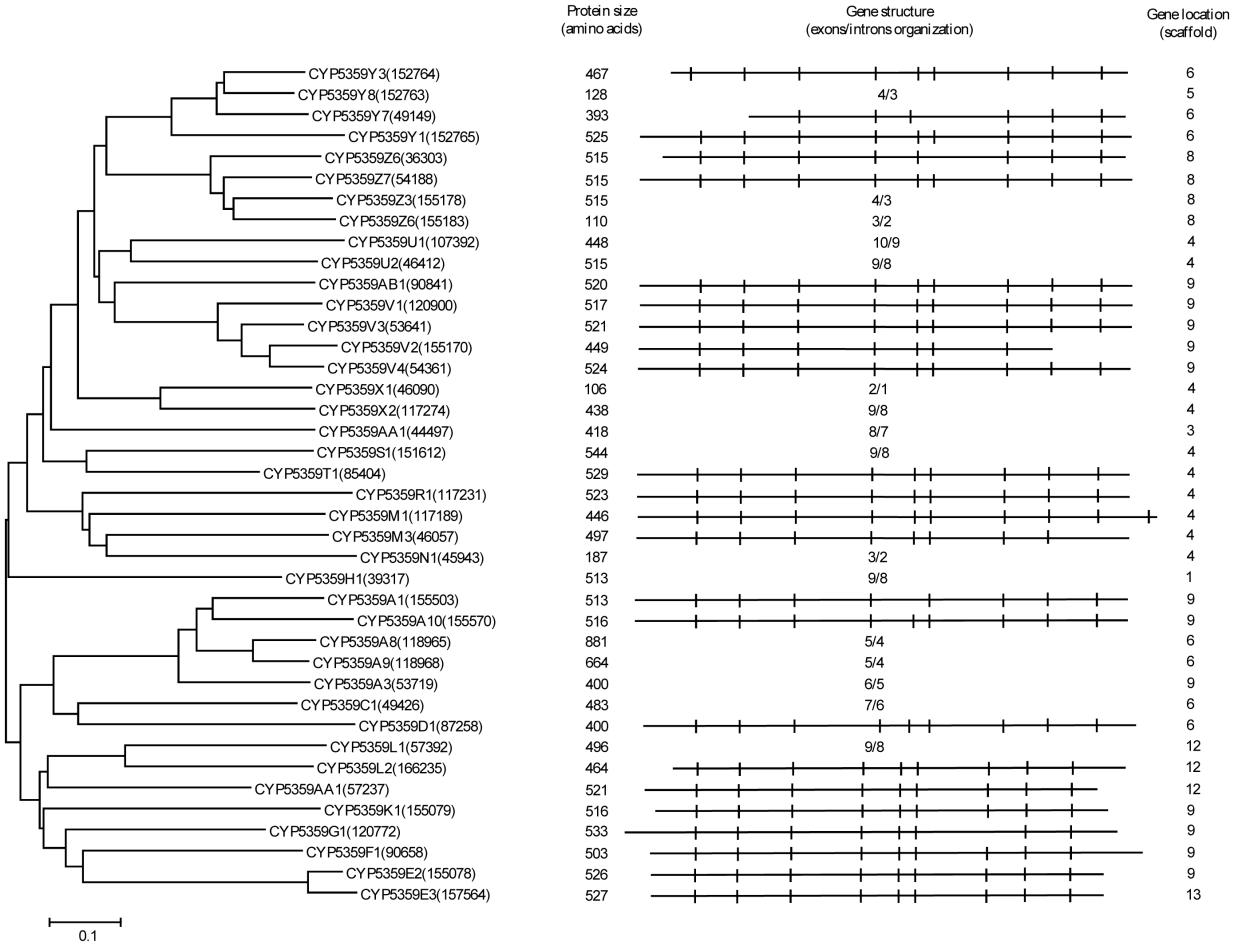
Phylogenetic and gene-structure analysis of CYP5359 family. Forty CYP5359 P450 sequences from *Ganoderma* sp. were included in the tree. A minimum evolution tree was constructed using the close-neighbor-interchange algorithm in MEGA (version 5.05). The protein size in amino acids is shown in the figure. The gene-structure analysis for each P450 was presented in the form of exon-intron organization. A graphical format showing parallel (gene size) and vertical lines (introns) is presented for P450s showing similar gene structure. For the rest of the P450s, the number of exons and introns was shown. The genetic location of P450 is shown in the form of the scaffold number.

In order to understand evolution of P450s in model basidiomycetes, we carried out gene-structure analysis on member P450s belonging to the enriched P450 family. The genetic location of P450s (Figures 2, 4–7, 9, 11, 12, S1–S3) across the model basidiomycetes suggested that the majority of the P450s belonging to the same family are tandemly arranged (located on the same scaffold) (Table 2). Tandem arrangement of P450s of the same family is an indication of possible gene duplication. Exon-intron organization of P450s revealed species-specific conservation in size of the exons and location of introns (Figures 2, 4–7, 9, 11, 12, S1–S3). This strongly suggests that P450s are extensively duplicated in model basidiomycetes after speciation (paralogs), possibly to cater to the need for adaptation to diverse ecological niches. The genetic location of duplicated P450s (Table 2) revealed tandem arrangement of a large number of duplicated P450s. Tandem gene duplication is a faster way of achieving large numbers of the same genes in order to adapt to ecological niches. Based on gene structure (exon-intron arrangement), we also identified a large number of ortholog P450s in the P450 families. In the CYP63 family, a large number of ortholog P450s were found between *Phanerochaete chrysosporium* and *Phanerochaete carnosa* (Fig. 2). A single ortholog P450 was found between model dry-rot fungus *Serpula lacrymans* (CYP63B, protein ID: 1150180) and model brown-rot fungus *Postia placenta* (CYP63B2, protein ID: 13867) (Fig. 2). In the CYP512 family a single ortholog P450 was found between *Phanerochaete chrysosporium* (CYP512E1, protein ID: 136973) and *Phanerochaete carnosa* (CYP512E, protein ID: 246674) (Fig. S1). In the CYP5035 family ortholog P450 of *Phanerochaete chrysosporium* (CYP5035C1, protein ID: 9198) was duplicated eight times in *Phanerochaete carnosa* (Fig. 4). A similar phenomenon was observed in the CYP5141 family, where P450 of *Phanerochaete chrysosporium*, CYP5144G1 (protein ID: 40563) is duplicated five times in *Phanerochaete carnosa* (Fig. S2). Exon-intron organization of CYP5150 family members (Fig. S3) showed high conservation in the size of exons, location of introns and number of introns in the genes across the model basidiomycete species (Table 2). This indicates that the majority of the CYP5150 family P450s originated before the speciation (orthologs). Gene-structure analysis showed extensive gene duplications of P450s in the P450 families CYP5348 (Fig. 11) and CYP5359 (Fig. 12); those are present in *Ganoderma* sp. and *Postia placenta* and only in *Ganoderma* sp. (Fig. 1).

**Table 2:** Analysis of P450 gene duplications and gene-structure (introns) in P450 families enriched in the basidiomycete species, *Phanerochaete chrysosporium*, *Phanerochaete carnosa*, *Agaricus bisporus*, *Ganoderma* sp., *Postia placenta*, and *Serpula lacrymans*.

P450 Family			basidiomycete						Figure
			*Phanerochaete chrysosporium*	*Phanerochaete carnosa*	*Agaricus bisporus*	*Ganoderma* sp.	*Postia placenta*	*Serpula lacrymans*	
**CYP63**	No. of P450s duplicated		7	8	3	3	4	7	Fig. 2
	Gene location^a^	Scaffolds	2,25,3,19	2,1,11	5,9	2	15,21,83	1,2,3,4,9,19	
		No. of P450s	3,1,2,1	3,4,1	1,2	3	2,1,1	2,1,1,1,1,1	
	Introns^b^	min-max	9–14	6–14	11–15	14–17	9–14	9–16	
		P/I	2/13	3/13	2/15;2/12;2/11	2/14	2/14;2/13	3/14;2/16	
**CYP512**	No. of P450s duplicated		8	12	9	17	6	9	Fig. S1
	Gene location	Scaffolds	4,14,18	1,2,6,8,9,13,657	2,14	1,3,5,8,10,11,16	8,48,98,	1,6,9,13,18	
		No. of P450s	1,1,6	4,1,1,2,2,1,1	8,1	5,1,2,3,2,2,2	2,1,3	3,2,1,1,2	
	Introns	min-max	7–10	8–11	9–11	7–11	8–18	9–13	
		P/I	5/10;5/8	6/9;3/10	5/10;3/14	14/10	7/10	7/14	
**CYP5035**	No. of P450s duplicated		2	8	FA	13	None	7	Fig. 4
	Gene location	Scaffolds	8,22	5,7,9,13,272		1,4,5,8,11,14,15		7	
		No. of P450s	1,1	1,1,3,2,1		3,1,1,3,2,1,2		2	
	Introns	min-max	6–10	6–12		7–9	7–15	NA	
		P/I	6/8	8/8		5/9;7/8	2/7	NA	
**CYP5037**	No. of P450s duplicated		None	3	2	3	10	8	Fig. 5
	Gene location	Scaffolds		4	4,10	10,11	26,45,65,100,135,163,206	4,8,9,14	
		No. of P450s		3	1,1	1,2	2,2,1,1,1,2,1	2,3,1,2	
	Introns	min-max	7–12	7–11	5–10	9–10	10–13	6–12	
		P/I	2/11	2/11	None	3/9;2/10	5/11;6/10	7/11	
**CYP5136**	No. of P450s duplicated		None	5	FA	8	FA	5	Fig. 6
	Gene location	Scaffolds		8,9,10		1,3,5		7	
		No. of P450s		2,2,1		1,4,3		5	
	Introns	min-max	9–14	13–15		13–14		14–15	
		P/I	2/13	3/14		7/13		4/15	
**CYP5141**	No. of P450s duplicated		None	2	4	None	None	5	Fig. 7
	Gene location	Scaffolds		8	12,13,17			2	
		No. of P450s		2	1,2,1			5	
	Introns	min-max	8–13	13–16	9–14	NA	15–32	7–18	
		P/I	2/12	2/15;2/14	2/11	NA	2/15	None	
**CYP5144**	No. of P450s duplicated		17	42	7	3	3	18	Fig. S2
	Gene location	Scaffolds	5,1,8,2,94	5,85,13,6,9,67,2,31135,	6,9,12,17,8,15	5	34,10,80	23,9,18,7,8,10,2,11	
		No. of P450s	1,10,2,3,1	29,2,1,1,3,1,1,3,1	1,1,2,1,1,1	3	1,1,1	2,1,1,6,3,3,1,1	
	Introns	min-max	8–13	9–27	7–11	9–12	9–12	8–13	
		P/I	11/13;6/11	12/13;13/11;7/10	4/11;5/9	NA	NA	9/11;8/10	
**CYP5146**	No. of P450s duplicated		2	12	FA	FA	FA	2	Fig. 9
	Gene location	Scaffolds	1	5,6,335				1	
		No. of P450s	2	4,7,1				2	
	Introns	min-max	9–13	9–12				NA	
		P/I	3/9	6/11;4/10				NA	
**CYP5150**	No. of P450s duplicated		3	9	12	33	28	2	Fig. S3
	Gene location	Scaffolds	2,8	2,4,13	17,13,7,18,3	12,1,5,4,14,8,11	Distributed on 22 scaffolds	3	
		No. of P450s	2,1	4,1,4	1,2,7,1,1	8,1,2,2,13,1,6		2	
	Introns	min-max	11–12	11–14	8–17	10–14	10–20	NA	
		P/I	2/11	6/13	5/16;4/17	9/13;12/12;7/11	19/13	2/13	
**CYP5348**	No. of P450s duplicated		FA	FA	FA	3	18	FA	Fig. 11
	Gene location	Scaffolds				8	133,9,75,15,171,46,64,126,1,72,228		
		No. of P450s				3	3,1,1,5,1,1,1,1,1,1,2		
	Introns	min-max				11–12	9–21		
		P/I				3/12	6/12;14/11;6/10		
**CYP5359**	No. of P450s duplicated		FA	FA	FA	24	FA	FA	Fig. 12
	Gene location	Scaffolds				4,6,8,9,12,13			
		No. of P450s				4,4,2,11,2,1			
	Introns	min-max				4–10			
		P/I				17/9			

a Scaffolds and P450s present on the scaffold were separated by commas. For more information on tandem gene arrangements in scaffold, see figures.

b For each P450 family the minimum and maximum number (min-max) of introns observed in member P450s (≥400 amino acid length) were presented. A number of P450s showing the same number of introns in their gene-structure (P/I) are also presented in the table.

Abbreviations: FA, Family is absent in the fungus; NA, not applicable owing to the presence of single or double copies of P450 genes and P450s with short amino acid length.

A detailed analysis of the number of introns in members of the enriched P450 family showed a characteristic pattern (Table 2) that further supports extensive duplication of P450 genes in basidiomycetes. We performed thorough analysis of the size range of the introns and number of P450s containing the same number of introns in enriched P450 families (Table 2). It is noteworthy that member P450s of enriched families showed selective enrichment for the number of introns in their gene structure; for example, 13 introns in P450 families CYP63 and CYP5150; eight introns in CYP5035 family; 10 introns in CYP512 family; 11 and 10 introns in CYP5037 family; 13 to 15 introns in CYP5136 family; 11 introns in CYP5144 family and 12 introns in CYP5348 family (Table 2).

In the light of the above results, we conclude that extensive gene duplications, particularly tandem gene duplication of P450s, led enrichment of these P450 families in the selected model basidiomycetes, possibly in response to ecological adaptation. Functional analysis of P450s will provide an answer on the physiological significance for enrichment of certain P450 families in these model basidiomycetes.

### Members of Enriched P450 Families are Catalytically Versatile and show Extraordinary Substrate Oxidation Capabilities

**Figure 13:**
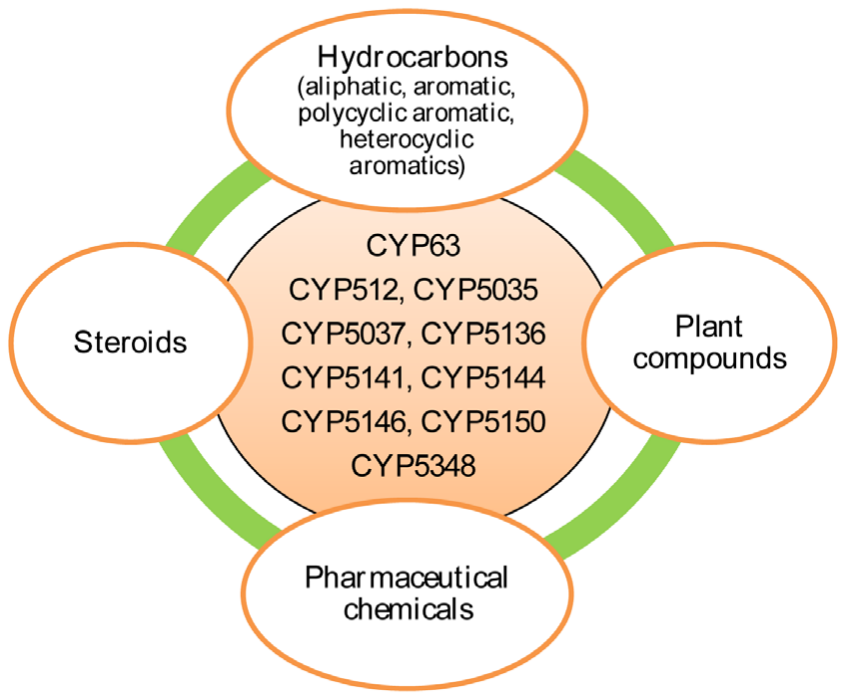
Catalytic versatility of members of P450 families enriched in selected six model basidiomycete fungi. Member P450s of enriched P450 families (shown in the center) are found to be catalytically versatile and show oxidation of different classes of compounds i.e. hydrocarbons, plant compounds, pharmaceutical chemicals and steroids. A detailed list on substrate specificity of enriched P450 family members is given in Table S3.

Enrichment of certain P450 families (identified in this study) in the selected model basidiomycete species suggests that these enriched P450 family proteins play a key role in fungal physiology (primary or secondary metabolism). Considering the large number of P450 proteins used in this study and the availability of functional data on fungal P450s [20], [34]–[42], we conducted an extensive literature review to reveal the substrate and catalytic specificities of P450 proteins belonging to the enriched families. Our analysis demonstrated that P450s belonging to the enriched families are catalytically versatile and show broad substrate specificity (Fig. 13 and Table S3). As shown in Fig. 13, a range of substrates belonging to different classes of compounds, such as hydrocarbons, steroids, plant compounds and pharmaceutical chemicals, were oxidized by member P450s of the enriched P450 family. Details on the catalytic versatility of member P450s of the enriched family are presented in Table S3. In a recent study, the CYP63 family P450 from *Phanerochaete chrysosporium* showed catalytic versatility and oxidized a range of xenobiotic compounds, such as hydrocarbons including alkanes, polycyclic aromatic compounds and alkylphenols [37]. The CYP63 family’s P450 oxidation capabilities were found to be superior compared to P450s across the biological kingdoms (bacteria and animals) [37]. Furthermore, the CYP63 family P450 from *Postia placenta* was found to oxidize heterocyclic aromatics [20].

CYP512 family member P450s showed broad substrate specificity and oxidized polycyclic aromatics [38], heterocyclic aromatics, plant compounds (resins) and steroids [41] (Table S3). A recent study describing the genome sequencing and transcriptome analysis of the medicinal mushroom, *Ganoderma lucidum* [9], revealed up-regulation of CYP512 family member P450s during synthesis of steroid lanosterol. Based on the oxidation of steroids (testosterone) [41] and up-regulation during synthesis of lanosterol, it is hypothesized that CYP512 family proteins may be involved in triterpenoids biosynthesis in *Ganoderma lucidum* [9]. Oxidation of plant defense chemicals such as resins by CYP512 [20] further suggests that CYP512 family members play a key role in detoxification of plant defense chemicals and possibly help fungi to colonize on wood. CYP5035 family proteins were found to oxidize plant chemicals, i.e. resin and flavonoids and the pharmaceutical chemical naproxen [41] (Table S3). Among the enriched families, member P450s belonging to the CYP5136, CYP5141 and CYP5150 families displayed diverse catalytic activities and oxidized hydrocarbons, plant chemicals, steroids and pharmaceuticals (Table S3). This clearly suggests that CYP5136, CYP5141 and CYP5150 family member P450s play a key role both in xenobiotic compounds oxidation and fungal primary and secondary metabolism. Proteins of the CYP5144 family, the largest P450 contingent family across the selected model basidiomycetes, showed oxidation of hydrocarbons, steroids and pharmaceuticals (Table S3). Based on up-regulation of CYP5144 P450s during synthesis of lanosterol steroid by *Ganoderma lucidum* [9], it is presumed that CYP5144 proteins were involved in fungal steroid metabolism. CYP5146 and CYP5348 family members showed oxidation of hydrocarbons including polycyclic aromatic compounds. However, compared to CYP5348 family proteins, CYP5146 family proteins showed further oxidation of heterocyclic aromatic compounds (Table S3). No data on functional analysis of the CYP5359 family, a newly identified family in *Ganoderma* sp., is available. However, the presence and enrichment of the CYP5359 family only in *Ganoderma* strains, *Ganoderma* sp. [12] and *Ganoderma lucidum* [9], suggest that CYP5359 family member P450s may be involved in the generation of bio-active compounds produced by this medicinal mushroom fungus.

### Role of Enriched P450 Families in Fungal Colonization of Wood

Enrichment of specific P450 families (Figure 1) in the selected model basidiomycete species showing preferences for degradation of different plant material (Table 1) suggested that member P450s belonging to these enriched families have a common function, possibly in fungal adaptation to diverse ecological niches by involvement in oxidation of plant material. Here, we present two types of evidence in support of the hypothesis. Oxidation of plant defense chemicals such as resins and coumarin by member P450s of enriched families [20], [41] (Table S3) indicates (direct evidence) that P450s are involved in detoxification of plant defense chemicals. Detoxification of plant defense chemicals is crucial in colonization of fungus on wood material. Indirect evidence on P450 involvement in plant material degradation can be obtained from P450 gene up-regulation studies [53]–[58]. Transcriptome analysis studies using the basidiomycetes, *Phanerochaete chrysosporium*, *Phanerochaete carnosa* and *Postia placenta,* showed up-regulation of member P450s that belong to enriched families during the growth of fungus on wood components or under growth conditions similar to fungal wood colonization. Growth of *Phanerochaete chrysosporium* under ligninolytic conditions showed up-regulation of CYP63 [53] and up-regulation of CYP5035 was observed under ligninolytic conditions supplemented with either glucose [55] or cellulose [54], [56]. Growth of *Phanerochaete carnosa* on wood resulted in up-regulation of 21 P450s [58]. Among the 21 P450s up-regulated, 17 P450s belonged to enriched families such as CYP5037 (one P450), CYP5144 (six P450s), CYP5036 (one P450), CYP63 (one P450), CYP5141 (three P450s), CYP512 (two P450s) and CYP5150 (three P450s) (the number of P450s found up-regulated in member P450 families is shown in parenthesis). Furthermore, up-regulation of CYP5150 (three P450s) and CYP512 (single P450) family members was observed during *Postia placenta* growth on wood material [57].

Based on the above data, we hypothesize that basidiomycete P450s, particularly members of an enriched P450 family, play key roles in fungal colonization of wood *via* either detoxification of plant defense chemicals or degradation of compounds derived from plant components (lignin, cellulose or hemicellulose). Further support for our hypothesis can be found in the fact that none of the 11 enriched P450 families were found in non-wood-degrading basidiomycetes (Table S2) strongly indicating the role of the members of the enriched P450 families in fungal colonization of wood. However, the specific catalytic roles of P450s up-regulated in model basidiomycetes are yet to be elucidated.

## Conclusions

The presence of a large contingent of P450s in fungi, particularly wood-degrading basidiomycetes, implies their important role in fungal metabolism and adaptation to diverse ecological niches. Following Sir Charles Darwin’s theory of natural selection, we performed genome-wide comparative P450 analysis and evolutionary analysis of P450 families to identify specific P450 families enriched in model basidiomycetes and to understand the molecular basis for selective P450 family enrichment. Our analysis showed enrichment of 11 P450 families out of 68 P450 families found in the basidiomycetes, *Phanerochaete chrysosporium*, *Phanerochaete carnosa*, *Agaricus bisporus*, *Postia placenta*, *Ganoderma* sp. and *Serpula lacrymans*. Evolutionary analysis on member P450s of enriched families suggested that extensive gene duplications, including tandem gene duplications, led to the enrichment of these families in model basidiomycetes. Functional analysis and gene up-regulation data suggested that the enriched P450 families of model basidiomycetes had a common physiological function, i.e. degradation of plant defense chemicals and plant-material-derived compounds, especially lignin derived compounds. It is noteworthy that members of enriched P450 families are catalytically diverse and show complement function. To our knowledge, this study is the first of its kind on the identification P450 families enriched in model basidiomycetes and comparative evolutionary analysis of representative P450s from an enriched family.

## Supporting Information

Figure S1 **Gene-structure analysis of CYP512 family.** In total 82 CYP512 P450 sequences from six model basidiomycetes (Fig. 1) were included in the tree. The minimum evolution tree was constructed using the close-neighbor-interchange algorithm in MEGA (version 5.05). For ease of visual identity, the tree branch color, protein name, protein ID (parenthesis) and model basidiomycete species name were presented with unique color. The protein size in amino acids is also shown in the figure. The gene-structure analysis for each P450 was presented in the form of exon-intron organization. A graphical format showing parallel (gene size) and vertical lines (introns) is presented for P450s showing similar gene structure (highlighted with unique back ground color). For the rest of the P450s, the number of exons and introns was shown. The genetic location of P450 is shown in the form of the scaffold number. Abbreviations: Pchr, *Phanerochaete chrysosporium*; Pcar, *Phanerochaete carnosa*; Abis, *Agaricus bisporus*; Gsp, *Ganoderma* sp.; Ppla, *Postia placenta*; Slac, *Serpula lacrymans*.
(PDF)

Figure S2 **Gene-structure analysis of CYP5144 family.** Gene-structure analysis for each P450 was presented in the form of exon-intron organization. A graphical format showing parallel (gene size) and vertical lines (introns) is presented for P450s showing similar gene structure (also highlighted with unique background color). For the rest of the P450s, the number of exons and introns was shown. For ease of visual identity, the P450 name, protein ID (parenthesis) and model basidiomycete species name were presented with unique color. The protein size in amino acids and genetic location of P450 in the form of the scaffold number are shown in the figure.
(PDF)

Figure S3 **Gene-structure analysis of CYP5150 family.** Gene-structure analysis for each P450 was presented in the form of exon-intron organization. A graphical format showing parallel (gene size) and vertical lines (introns) is presented for P450s showing similar gene structure. For the rest of the P450s, the number of exons and introns was shown. For ease of visual identity, the P450 name, protein ID (parenthesis) and model basidiomycete species name were presented with unique color. The protein size in amino acids and genetic location of P450 in the form of scaffold number are shown in the figure.
(PDF)

Table S1 **Comparative analysis of P450 monooxygenases in basidiomycete species,** ***Phanerochaete chrysosporium*** **(Pchr),** ***Phanerochaete carnosa*** **(Pcar),** ***Agaricus bisporus*** **(Abis),** ***Ganoderma*** **sp. (Gsp),** ***Postia placenta*** **(Ppla), and** ***Serpula lacrymans*** **(Slac).**
(PDF)

Table S2 **Comparative analysis of P450 monooxygenases between** ***Tremella mesenterica*** **(mycoparasite) and** ***Cryptococcus neoformans*** **(animal pathogen/parasite).**
(PDF)

Table S3 **Catalytic versatility of members of P450 families enriched in basidiomycete species,** ***Phanerochaete chrysosporium*** **,** ***Phanerochaete carnosa*** **,** ***Agaricus bisporus*** **,** ***Ganoderma sp./Ganoderma lucidum*** **,** ***Postia placenta*** **and** ***Serpula lacrymans*** **^a^.**
(PDF)
