# Supplementary figures and images for: Systematic Identification and Evolutionary Analysis of Catalytically Versatile Cytochrome P450 Monooxygenase Families Enriched in Model Basidiomycete Fungi
Source: PLoS One. 2014 Jan 22;9(1):e86683. doi: 10.1371/journal.pone.0086683 (PMC3899305; doi:10.1371/journal.pone.0086683)

Figure S1

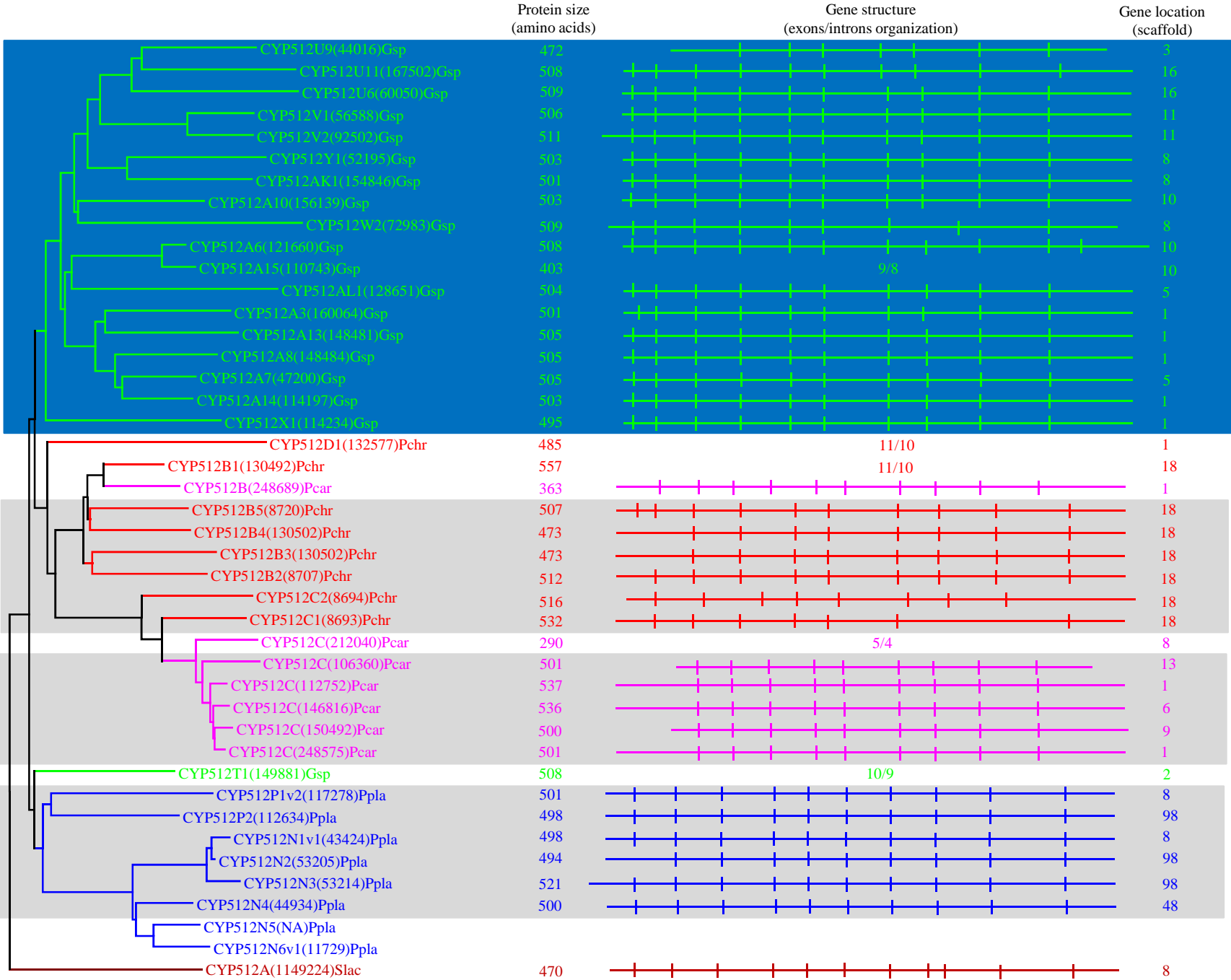

**Figure S1 continued**

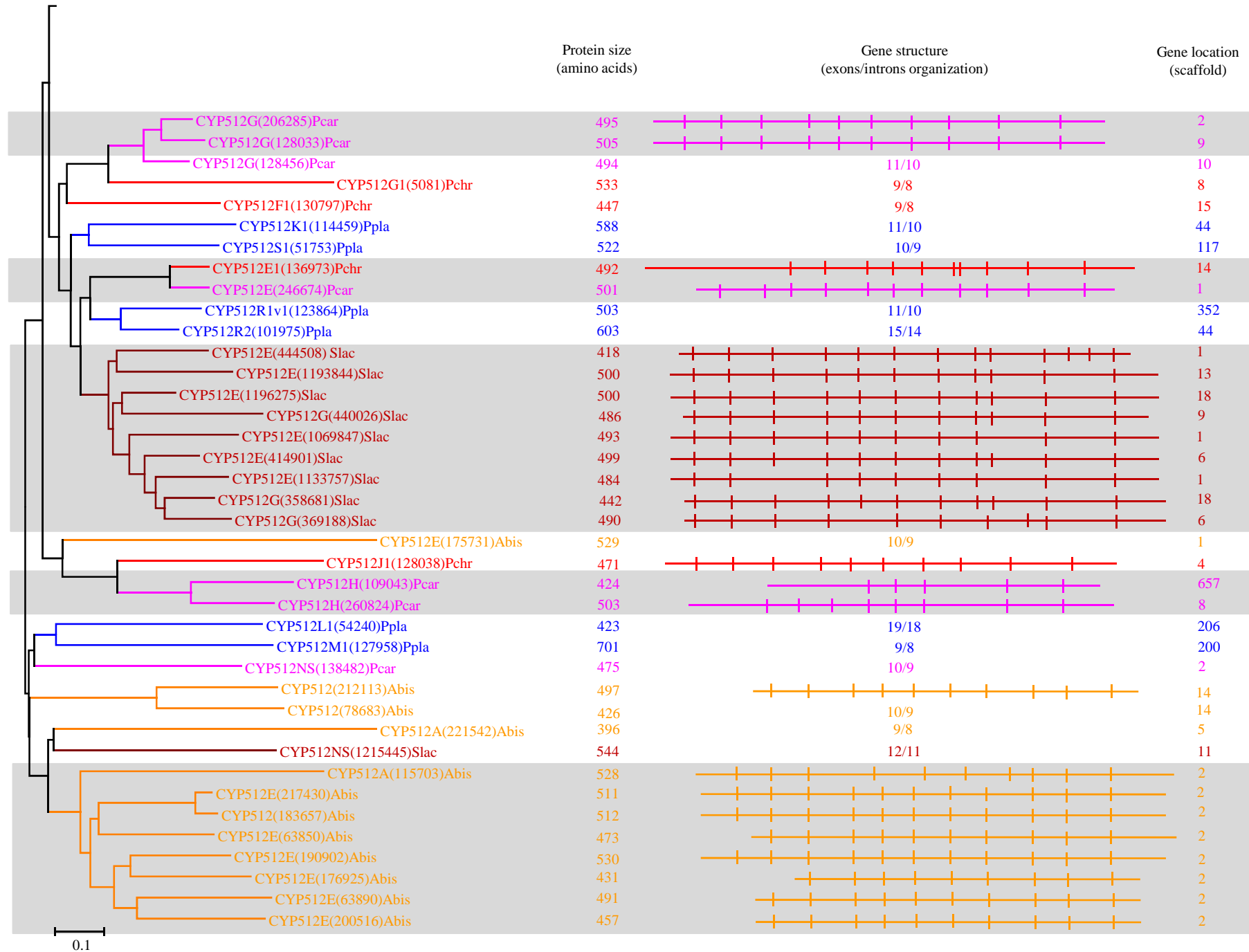

Supplement: Figure S1 — Gene-structure analysis of CYP512 family. In total 82 CYP512 P450 sequences from six model basidiomycetes (Fig. 1) were included in the tree. The minimum evolution tree was constructed using the close-neighbor-interchange algorithm in MEGA (version 5.05). For ease of visual identity, the tree branch color, protein name, protein ID (parenthesis) and model basidiomycete species name were presented with unique color. The protein size in amino acids is also shown in the figure. The gene-structure analysis for each P450 was presented in the form of exon-intron organization. A graphical format showing parallel (gene size) and vertical lines (introns) is presented for P450s showing similar gene structure (highlighted with unique back ground color). For the rest of the P450s, the number of exons and introns was shown. The genetic location of P450 is shown in the form of the scaffold number. Abbreviations: Pchr, Phanerochaete chrysosporium; Pcar, Phanerochaete carnosa; Abis, Agaricus bisporus; Gsp, Ganoderma sp.; Ppla, Postia placenta; Slac, Serpula lacrymans. (PDF) [file pone.0086683.s001.pdf]
