# Supplementary material for: Systematic Identification and Evolutionary Analysis of Catalytically Versatile Cytochrome P450 Monooxygenase Families Enriched in Model Basidiomycete Fungi
Source: PLoS One. 2014 Jan 22;9(1):e86683. doi: 10.1371/journal.pone.0086683 (PMC3899305; doi:10.1371/journal.pone.0086683)
Supplement: Figure S2 — Gene-structure analysis of CYP5144 family. Gene-structure analysis for each P450 was presented in the form of exon-intron organization. A graphical format showing parallel (gene size) and vertical lines (introns) is presented for P450s showing similar gene structure (also highlighted with unique background color). For the rest of the P450s, the number of exons and introns was shown. For ease of visual identity, the P450 name, protein ID (parenthesis) and model basidiomycete species name were presented with unique color. The protein size in amino acids and genetic location of P450 in the form of the scaffold number are shown in the figure. (PDF) [file pone.0086683.s002.pdf]

Figure S2

	Protein size (amino acids)	Gene structure (exons/introns organization)	Gene location (scaffold)
CYP5144C(1217614)Slac	457		23
CYP5144C(1214969)Slac	545		9
CYP5144C(1217615)Slac	492		23
CYP5144C(1190676)Slac	491	9/8	9
CYP5144A(1063792)Slac	271	8/7	9
CYP5144C(439936)Slac	494	11/10	9
CYP5144NS(350327)Slac	494	9/8	9
CYP5144C(449580)Slac	527	14/13	7
CYP5144C(453964)Slac	520		18
CYP5144C(1123316)Slac	439		7
CYP5144C(416169)Slac	485		8
CYP5144C(1151506)Slac	492		10
CYP5144C(1190901)Slac	517		10
CYP5144C(1204244)Slac	484		8
CYP5144C(409113)Slac	515		8
CYP5144C(365613)Slac	491		2
CYP5144C(1205428)Slac	476		10
CYP5144C(449501)Slac	517		7
CYP5144C(440764)Slac	493		11
CYP5144C(1213798)Slac	486		7
CYP5144C(1213831)Slac	520		7
CYP5144C(468716)Slac	553		7
CYP5144C(349289) Slac	487		7
CYP5144(180182)Abis	514	10/9	9
CYP5144C(179058)Abis	518		6
CYP5144C(225715)Abis	517		9
CYP5144D(226079)Abis	469	10/9	9
CYP5144C(213191)Abis	506	11/10	19
CYP5144C(188118)Abis	509		12
CYP5144C(121940)Abis	458		12
CYP5144C(210512)Abis	240	7/6	11
CYP5144C(79606)Abis	446	6/7	17
CYP5144C(227934)Abis	532	14/13	12
CYP5144C(228060)Abis	491	9/8	12
CYP5144C(212894)Abis	521		17
CYP5144C(186880)Abis	520		8
CYP5144C(212451)Abis	520		15

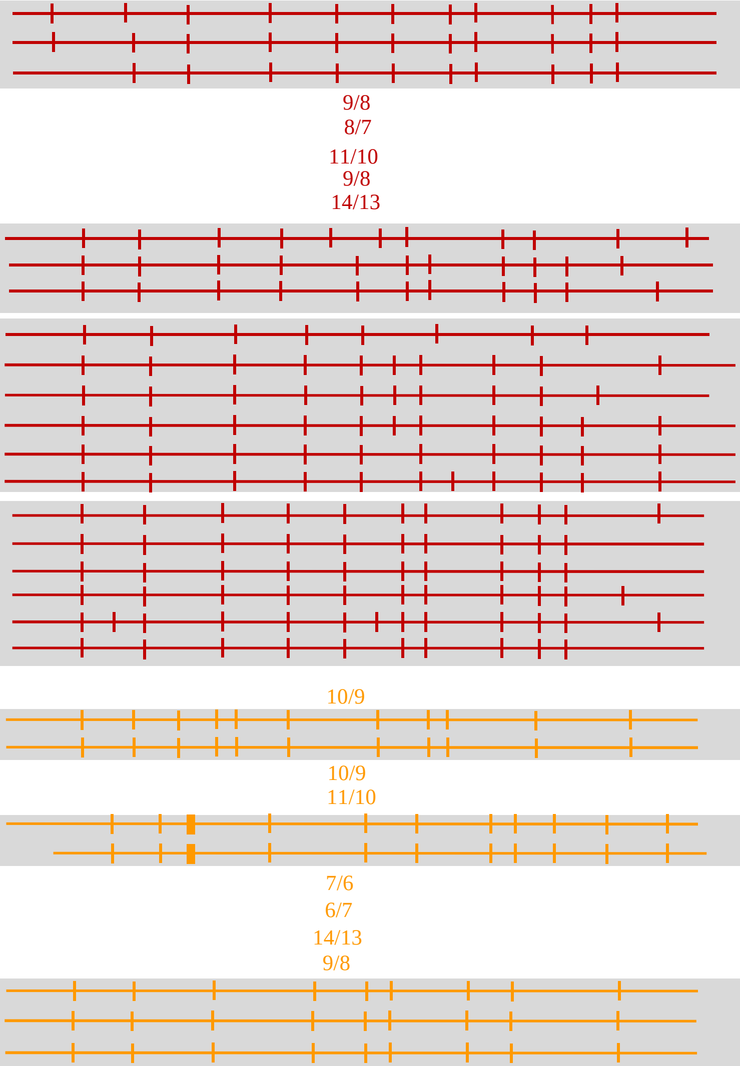

Figure S2 continued

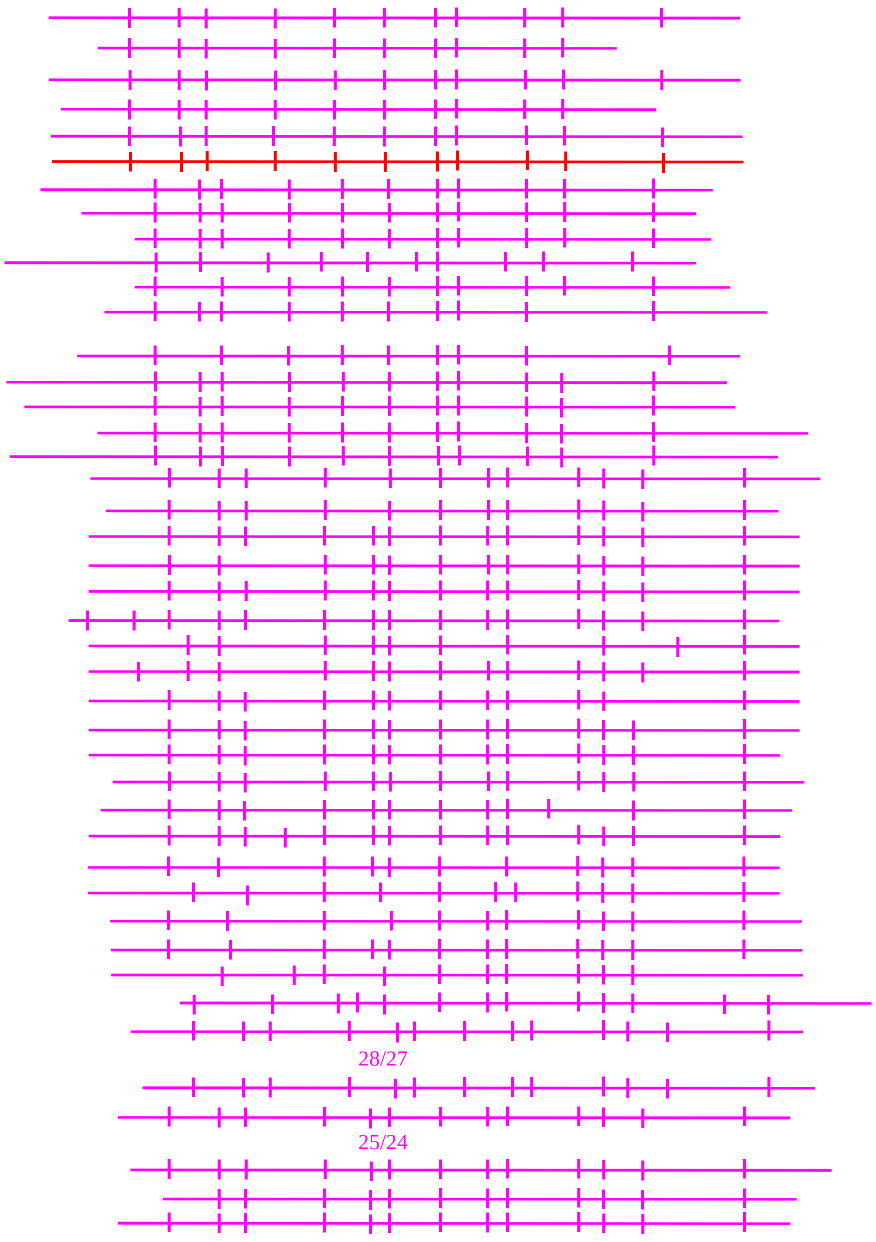

	Protein size (amino acids)	Gene structure (exons/introns organization)	Gene location (scaffold)
CYP5144G(208500)Pcar	529		5
CYP5144G(215005)Pcar	425		1135
CYP5144G(208499)Pcar	529		5
CYP5144G(194981)Pcar	500		5
CYP5144G(208500)Pcar	529		5
CYP5144G1(40563)Pchr	521		5
CYP5144F(119876)Pcar	517		5
CYP5144F(208527)Pcar	515		5
CYP5144F(142803)Pcar	467		5
CYP5144H(255191)Pcar	574		5
CYP5144H(255217)Pcar	465		5
CYP5144F(214509)Pcar	543		85
CYP5144H(142793)Pcar	526		5
CYP5144H(214508)Pcar	553		85
CYP5144H(142788)Pcar	528		5
CYP5144H(142748)Pcar	505		5
CYP5144H(208506)Pcar	532		5
CYP5144E(265238)Pcar	451		13
CYP5144A(96498)Pcar	520		6
CYP5144A(144742)Pcar	518		5
CYP5144A(256793)Pcar	395		5
CYP5144A(209385)Pcar	516		5
CYP5144A(209392)Pcar	739		5
CYP5144A(195925)Pcar	485		5
CYP5144A(256807)Pcar	409		5
CYP5144A(256813)Pcar	514		5
CYP5144A(209390)Pcar	476		5
CYP5144A(209396)Pcar	517		5
CYP5144A(256800)Pcar	510		5
CYP5144A(209397)Pcar	485		5
CYP5144A(209389)Pcar	502		5
CYP5144D(144778)Pcar	515		5
CYP5144D(121872)Pcar	512		5
CYP5144B(150298)Pcar	504		9
CYP5144B(212190)Pcar	496		9
CYP5144B(176971)Pcar	372		9
CYP5144B(265797)Pcar	394		67
CYP5144C(249435)Pcar	515		2
CYP5144C(256765)Pcar	511	28/27	5
CYP5144C(209373)Pcar	509		5
CYP5144C(209340)Pcar	524		5
CYP5144C(256701)Pcar	510	25/24	5
CYP5144C(252949)Pcar	433		3
CYP5144C(253005)Pcar	409		3
CYP5144C(207382)Pcar	511		3
CYP5144C(89562)Pcar	443	8/7	3

Figure S2 continued

	Protein size (amino acids)	Gene structure (exons/introns organization)	Gene location (scaffold)
CYP5144E1(8627)Pchr	400	9/8	17
CYP5144A10(38487)Pchr	469		1
CYP5144A8(787)Pchr	547		1
CYP5144A2(38024)Pchr	468	14/13	1
CYP5144A7(133311)Pchr	510		1
CYP5144A4(132481)Pchr	488	11/10	1
CYP5144A3(133327)Pchr	516		1
CYP5144A12(5055)Pchr	530		8
CYP5144A13(5054)Pchr	524		8
CYP5144A18(782)Pchr	592	8/7	1
CYP5144A11(37971)Pchr	480		1
CYP5144A5(132481)Pchr	488	14/13	1
CYP5144A6(785)Pchr	346	7/6	1
CYP5144D3(133291)Pchr	510		1
CYP5144D2(133482)Pchr	520		1
CYP5144D1(132401)Pchr	515		1
CYP5144D4(132914)Pchr	487	12/11	1
CYP5144D5(798)Pchr	498	9/8	1
CYP5144J1(133273)Pchr	493	15/14	1
CYP5144B1(138753)Pchr	382		1
CYP5144C2(761)Pchr	429	11/10	1
CYP5144C6(139146)Pchr	512		2
CYP5144C3(1707)Pchr	433		2
CYP5144C5(139870)Pchr	527	14/13	2
CYP5144C4(139337)Pchr	513		2
CYP5144C1(766)Pchr	496		1
CYP5144C7(131921)Pchr	516		9
CYP5144C8(138183)Pchr	488	12/11	4
CYP5144H1(38348)Pchr	452	10/9	5
CYP5144L1(54688)Ppla	518		34
CYP5144K1v1(105726)Ppla	520		10
CYP5144K1v2(45371)Ppla	512		80
CYP5144N2(72322)Gsp	452		5
CYP5144L2(53308)Gsp	452		5
CYP5144N1(53175)Gsp	452		5
