# Supplementary material for: Systematic Identification and Evolutionary Analysis of Catalytically Versatile Cytochrome P450 Monooxygenase Families Enriched in Model Basidiomycete Fungi
Source: PLoS One. 2014 Jan 22;9(1):e86683. doi: 10.1371/journal.pone.0086683 (PMC3899305; doi:10.1371/journal.pone.0086683)

Figure S3

	Protein size (amino acids)	Gene location (scaffold)
CYP5150D8(92219)Ppla	554	181
CYP5150D9(92220)Ppla	546	181
CYP5150D5(56431)Ppla	548	31
CYP5150D16v1(58496)Ppla	539	38
CYP5150D16v2(89777)Ppla	527	81
CYP5150D7(56490)Ppla	552	31
CYP5150D1(56521)Ppla	548	31
CYP5150D6(107860)Ppla	231	31
CYP5150D4(56515)Ppla	546	31
CYP5150D20(46717)Ppla	533	41
CYP5150D2(56458)Ppla	451	31
CYP5150D18(21309)Ppla	472	107
CYP5150D17v1(97505)Ppla	578	14
CYP5150D17v2(97728)Ppla	1054	65
CYP5150D19(57109)Ppla	562	9
CYP5150D21(62768)Ppla	508	75
CYP5150D14v1(44177)Ppla	493	20
CYP5150D15v1(120723)Ppla	547	46
CYP5150D15v2(116443)Ppla	500	238
CYP5150D11v1(55398)Ppla	544	142
CYP5150D11v2(89660)Ppla	540	21
CYP5150D12v1(114266)Ppla	543	177
CYP5150D13v1(20385)Ppla	526	96
CYP5150D13v2(51235)Ppla	546	5
CYP5150D22(127929)Ppla	821	309
CYP5150F1(51017)Ppla	536	154
CYP5150E1v2(56875)Ppla	535	27
CYP5150E1v1(94692)Ppla	598	33

Gene structure
(exons/introns organization)

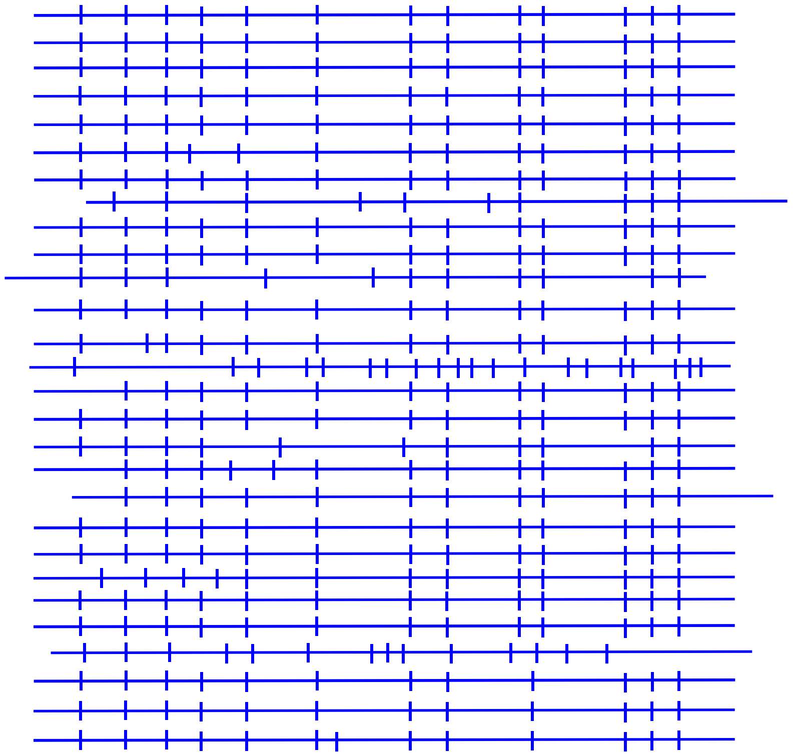

Figure S3 continued

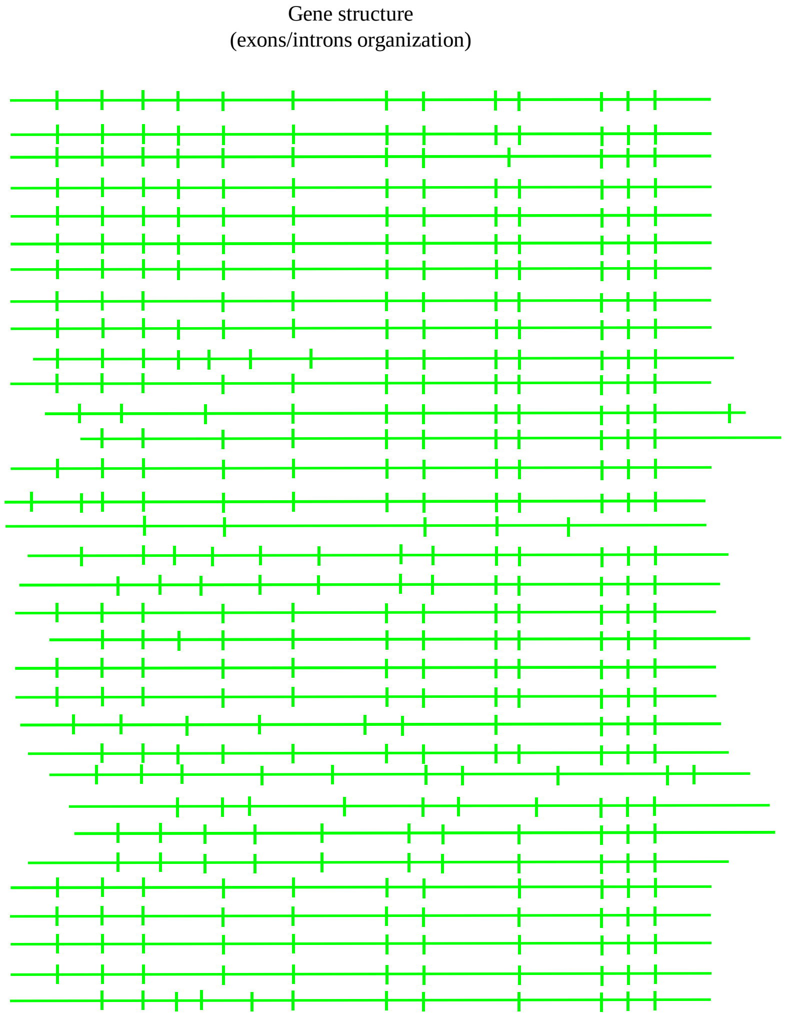

	Protein size (amino acids)	Gene location (scaffold)
CYP5150D25(166498)Gsp	549	12
CYP5150D24(157234)Gsp	547	12
CYP5150D23(56993)Gsp	555	12
CYP5150D27(147311)Gsp	541	1
CYP5150D29(122823)Gsp	545	12
CYP5150D28(157358)Gsp	564	12
CYP5150M5(152188)Gsp	548	5
CYP5150M(46275)Gsp	552	4
CYP5150G1(146207)Gsp	538	14
CYP5150H1(90218)Gsp	528	8
CYP5150J17(59629)Gsp	543	14
CYP5150J18(123794)Gsp	467	14
CYP5150J16(123793)Gsp	467	14
CYP5150J19(59491)Gsp	543	14
CYP5150J5(94625)Gsp	544	14
CYP5150J12(122158)Gsp	205	11
CYP5150J11(122142)Jsp	545	11
CYP5150J13(122244)Gsp	545	11
CYP5150J14(158128)Gsp	546	14
CYP5150J15(158130)Gsp	604	14
CYP5150K2(59427)Gsp	547	14
CYP5150K4(59616)Gsp	548	14
CYP5150L8(59501)Gsp	516	14
CYP5150L9(123691)Gsp	542	14
CYP5150L7(47827)Gsp	539	5
CYP5150L6(123698)Gsp	546	14
CYP5150L1(92661)Gsp	540	11
CYP5150L3(166075)Gsp	549	11
CYP5150L2(46997)Gsp	550	4
CYP5150L4(156784)Gsp	543	11
CYP5150L10(55691)Gsp	539	12
CYP5150L11(56190)Gsp	546	12
CYP5150E2(122651)Gsp	598	12

Figure S3 continued

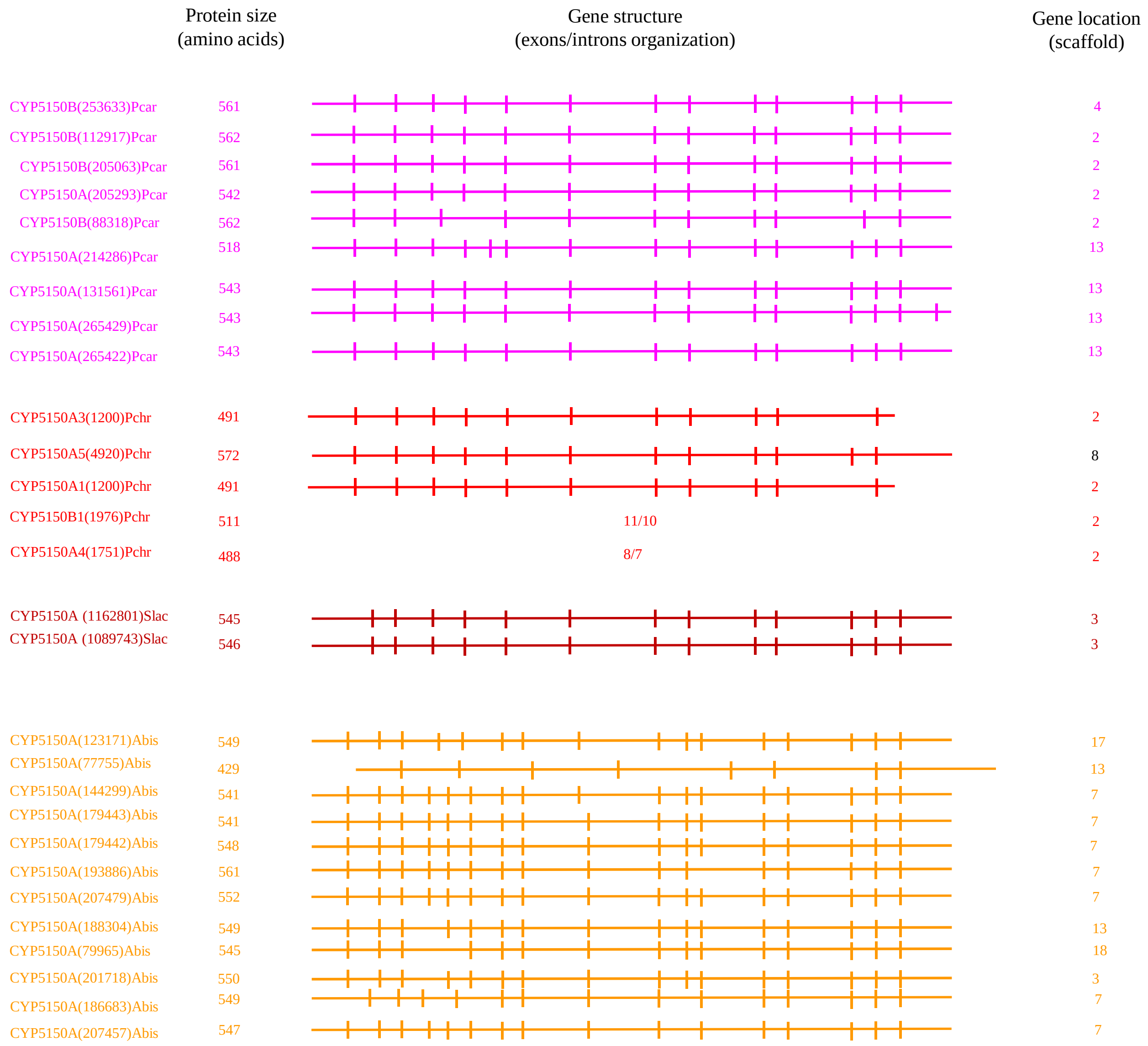

Supplement: Figure S3 — Gene-structure analysis of CYP5150 family. Gene-structure analysis for each P450 was presented in the form of exon-intron organization. A graphical format showing parallel (gene size) and vertical lines (introns) is presented for P450s showing similar gene structure. For the rest of the P450s, the number of exons and introns was shown. For ease of visual identity, the P450 name, protein ID (parenthesis) and model basidiomycete species name were presented with unique color. The protein size in amino acids and genetic location of P450 in the form of scaffold number are shown in the figure. (PDF) [file pone.0086683.s003.pdf]
