# Supplementary material for: Systematic Identification and Evolutionary Analysis of Catalytically Versatile Cytochrome P450 Monooxygenase Families Enriched in Model Basidiomycete Fungi
Source: PLoS One. 2014 Jan 22;9(1):e86683. doi: 10.1371/journal.pone.0086683 (PMC3899305; doi:10.1371/journal.pone.0086683)
Supplement: Table S1 — Comparative analysis of P450 monooxygenases in basidiomycete species, Phanerochaete chrysosporium (Pchr), Phanerochaete carnosa (Pcar), Agaricus bisporus (Abis), Ganoderma sp. (Gsp), Postia placenta (Ppla), and Serpula lacrymans (Slac). (PDF) [file pone.0086683.s004.pdf]

P450 family	Pchr	Pcar	Abis	Gsp	Ppla	Slac	P450 count	
							Number	Percentage[a]
CYP51	1	2	1	1	1	1	7	0.66
CYP53	1	7	2	1	7	1	19	1.79
CYP61	1	1	1	1	1	2	7	0.66
CYP63	7	9	6	5	5	7	39	3.68
CYP502	1	1	1	6	4	2	15	1.41
CYP504						1	1	0.09
CYP505	7	4		3	2		16	1.51
CYP512	14	27	12	18	14	11	96	9.05
CYP526						1	1	0.09
CYP530			1				1	0.09
CYP537				1	2		3	0.28
CYP548						1	1	0.09
CYP553						1	1	0.09
CYP613						3	3	0.28
CYP620		1	2			4	7	0.66
CYP634						1	1	0.09
CYP642				1			1	0.09
CYP645						1	1	0.09
CYP661						4	4	0.38
CYP5025		1				1	2	0.19
CYP5027					9		9	0.85
CYP5032			3			1	4	0.38
CYP5035	13	14		13	3	3	46	4.34

CYP5036	5	8				1	14	1.32
CYP5037	5	8	5	5	13	18	54	5.09
CYP5065			3	1		2	6	0.57
CYP5068			1				1	0.09
CYP5082						1	1	0.09
CYP5136	5	8		9		5	27	2.54
CYP5137	2	4	1	1	6	6	20	1.89
CYP5138	1	2		1	1	1	6	0.57
CYP5139	1	11	3	6	8	1	30	2.83
CYP5140	1	1	1	1	1	1	6	0.57
CYP5141	7	9	7	2	4	5	34	3.2
CYP5142	7	8	1			2	18	1.7
CYP5143	2	2				2	6	0.57
CYP5144	34	71	43	3	3	29	183	17.2
CYP5145	3	2	1			1	7	0.66
CYP5146	6	15				2	23	2.17
CYP5147	6	7					13	1.23
CYP5148	2	7	2	3	1	1	16	1.51
CYP5149	1	3			1		5	0.47
CYP5150	7	10	12	33	23	2	87	8.2
CYP5151	1	2	1	1	1	3	9	0.85
CYP5152	2	4		1	2	12	21	1.98
CYP5153		1	1				2	0.19
CYP5154	1	4				5	10	0.94
CYP5155	1	1					2	0.19
CYP5156	2	1	1	1	1	5	11	1.04
CYP5157	1	1				1	3	0.28

CYP5158	1	5		1	2		9	0.85
CYP5339					2		2	0.19
CYP5340				3	1		4	0.38
CYP5341				2	3		5	0.47
CYP5342					1		1	0.09
CYP5343					1		1	0.09
CYP5344					3		3	0.28
CYP5346					1		1	0.09
CYP5347				1	2		3	0.28
CYP5348				4	34		38	3.58
CYP5349				1	2		3	0.28
CYP5350					11		11	1.04
CYP5351				1	1		2	0.19
CYP5352					1		1	0.09
CYP5353					1		1	0.09
CYP5354					2		2	0.19
CYP5355					1		1	0.09
CYP5356					1		1	0.09
CYP5357				2			2	0.19
CYP5358				3			3	0.28
CYP5359				40			40	3.77
CYP5360				1			1	0.09
CYP5361				2			2	0.19
CYP5362				1			1	0.09
CYP5363								0
CYP5364				4			4	0.38
CYP5365				1			1	0.09

CYP5366				1			1	0.09
CYP5445					1		1	0.09
CYP6001		4					4	0.38
CYP6005				2			2	0.19
NA			3			7	10	0.94
**P450 count**	**149**	**266**	**115**	**188**	**184**	**159**	**1061**	

[a], Percentage for each P450 family was calculated considering total P450 count (1061) as 100%.
