# Supplementary material for: Systematic Identification and Evolutionary Analysis of Catalytically Versatile Cytochrome P450 Monooxygenase Families Enriched in Model Basidiomycete Fungi
Source: PLoS One. 2014 Jan 22;9(1):e86683. doi: 10.1371/journal.pone.0086683 (PMC3899305; doi:10.1371/journal.pone.0086683)
Supplement: Table S2 — Comparative analysis of P450 monooxygenases between Tremella mesenterica (mycoparasite) and Cryptococcus neoformans (animal pathogen/parasite). (PDF) [file pone.0086683.s005.pdf]

P450 family	*Tremella mesenterica*	*Cryptococcus neoformans*
CYP5216	1	2
CYP61	1	2
CYP51	1	2
CYP5215	1	2
CYP615	2	
CYP531	1	
CYP5139	1	2
