# Supplementary material for: Systematic Identification and Evolutionary Analysis of Catalytically Versatile Cytochrome P450 Monooxygenase Families Enriched in Model Basidiomycete Fungi
Source: PLoS One. 2014 Jan 22;9(1):e86683. doi: 10.1371/journal.pone.0086683 (PMC3899305; doi:10.1371/journal.pone.0086683)
Supplement: Table S3 — Catalytic versatility of members of P450 families enriched in basidiomycete species, Phanerochaete chrysosporium , Phanerochaete carnosa , Agaricus bisporus , Ganoderma sp./Ganoderma lucidum , Postia placenta and Serpula lacrymans a. (PDF) [file pone.0086683.s006.pdf]

**CYP Family**	**Hydrocarbons**	**Plant compounds**	**Steroids**	**Pharmaceutical chemicals**
CYP63	Alkanes (C3-C9 & C15-C19); Polycyclic aromatics [pyrene, benzo(*a*)pyrene, benzo(*ghi*)perylene]; Alkylphenols (C3-C9); Heterocyclic aromatics (carbazole, 3,5,4'-Trimethoxy-*trans*-stilbene)	-	-	-
CYP512	Polycyclic aromatics (anthracene); Heterocyclic aromatics (dibenzothiophene[b])	Resins (dehydroabietic acid)	Testosterone<br>Progesterone	-
CYP5035	-	Resins (dehydroabietic acid); Flavonoids (flavone)	-	Naproxen
CYP5037	Polycyclic aromatics (anthracene)	Coumarins (7-ethoxycoumarin)	-	-
CYP5136	Aromatics (4-ethoxybenzoic acid)	Resins (dehydroabietic	Testosterone	Diclofenac, compactin,

	Polycyclic aromatics [naphthalene, fluorene, phenanthrene, pyrene, benzo(*a*)pyrene, biphenyl]; Alkylphenols (C3-C9); Heterocyclic aromatics (carbazole, dibenzofuran, dibenzothiophene, 3,5,4’-Trimethoxy-*trans*-stilbene);Polychlorinated dibenzo-*p*-dioxins (mono- and di)	acid)<br>Coumarins (7-ethoxycoumarin)<br>Flavonoids (flavone)	Progesterone	Naproxen
CYP5141	Polycyclic aromatics (naphthalene, anthracene, *trans*-stilbene); Heterocyclic aromatics (carbazole)	Resins (dehydroabietic acid)<br>Flavonoids (flavone)<br>Coumarins (7-ethoxycoumarin)	Testosterone<br>Progesterone	Naproxen
CYP5144	Polycyclic aromatics (biphenyl, anthracene, phenanthrene, pyrene, benzo(*a*)pyrene, 3,5-	-	Testosterone<br>Progesterone	Compactin

	dimethoxy-*trans*-stilbene, 3,5,4’-Trimethoxy-*trans*-stilben); Heterocyclic aromatics (carbazole, dibenzothiophene)			
CYP5146	Polycyclic aromatics (anthracene); Heterocyclic aromatics (mono-chlorinated dibenzo-*p*-dioxin)	-	-	-
CYP5150	Aromatics (4-propyl to 4-heptylbenzoic acid); Polycyclic aromatics (naphthalene, anthracene, phenanthrene, pyrene, fluorene, *trans*-stilbene, biphenyl); Heterocyclic aromatics (carbazole, dibenzofuran, dibenzothiophene)	Resins (dehydroabietic acid)<br>Flavonoids (flavone)<br>Coumarins (7-ethoxycoumarin)	Testosterone	Diclofenac, Naproxen
CYP5348	Polycyclic aromatics (phenanthrene)	-	-	-

[a], Functional data for member P450s in enriched P450 families were resourced from published literature (20, 34-42). For ease of comparison activities were presented at family level. CYP5359 family P450s were not tested for activity against any of the above compounds. Functional data for entire members of the P450 family are not given, hence for some P450 families a dash (-) was placed against xenobiotic compound activity.

[b], CYP512 performs *S*-hydroxylation, *S*-oxidation and 2-hydroxylation of dibenzothiophene.
